# Life satisfaction effects of national identity, global identity, and their interactions

**DOI:** 10.1038/s41598-025-29471-8

**Published:** 2025-12-08

**Authors:** Glen Spiteri, Seamus Kim, Falk Lieder

**Affiliations:** Department of Psychology, University of California, Los Angeles (UCLA), Los Angeles, USA

**Keywords:** National citizenship identification, Global citizenship identification, Identification with all humanity, National pride, Cosmopolitanism, Life satisfaction, Psychology, Human behaviour

## Abstract

Expansive social identifications—including with one’s nation and the world—have been increasingly linked to psychological well-being. However, research has yet to examine how these effects interact. Although national and global identification are often assumed to be in conflict, it remains unclear whether strongly holding both diminishes or enhances overall well-being. We investigate this gap using Waves 5 and 6 of the World Values Survey (WVS; *N* = 100,650, across developing and developed countries). First, we replicated prior findings via mixed models, finding that national citizenship and national pride (capturing national identity), as well as world citizenship (capturing global identity) each robustly predicted life satisfaction. We then tested interactions and estimated life satisfaction across different combinations of predictor strengths. Despite a negative interaction between national pride and world citizenship, those high on both still reported the highest life satisfaction. Our findings suggest that the joint effects on well-being of national and global identifications, despite their seemingly competing natures, remain additive. We point to the need for future research on the underlying psychological mechanisms and behavioral consequences of these joint identifications.

**Supplementary Information:**

The online version contains supplementary material available at 10.1038/s41598-025-29471-8.

## Introduction

Since the 1970s, globalization has rapidly accelerated across political, economic, and—of present focus—social dimensions^1^. Unprecedented connectivity has challenged the salience of longstanding identities grounded in geographic and political boundaries while giving way to new modes of social identification^2^. And yet, nation-based identities have proven not only persistent but, especially in recent years, highly consequential^3^. For scholars who defend national identification as an important source of self-esteem and psychological anchorage amid global change^4^, this continued prominence may come as no surprise. Indeed, the thesis that national identification promotes psychological well-being enjoys firm empirical footing in the social psychological literature (discussed below). Yet, at the same time, empirical findings suggest that global identification also promotes psychological well-being.

Theoretical accounts of these two superordinate identifications—national and global—often (at least, implicitly) suggest inverse, mutually exclusive mechanisms: national identity boosts psychological well-being via in-group/out-group differentiation, and global identity boosts psychological well-being through transcending parochial differentiation. If both are taken as true, a downstream speculation is that while national and global identification can independently promote psychological well-being, their effects are zero-sum when held together. However, drawing on other research on nested and multiple identities, we propose an alternative possibility: when held jointly, identifying with both one’s nation and the world can work synergistically in promoting psychological well-being. In this study, we first replicate previous findings that national identity—captured here independently as national citizenship and national pride—and global identity—captured by world citizenship—each independently promote life satisfaction (our measure of psychological well-being). Then, we test the effects on life satisfaction yielded by various combinations of these identities. The following sections summarize the theory and empirical evidence motivating our research.

Emerging in the late 20th century, social identity theory (SIT) not only directed substantial attention to the psychological importance of social identity but also established a widely influential account of its underlying mechanisms^5–8^. Central to this account is the “self-esteem hypothesis”^9^: individuals are driven to identify with social groups as a means of creating a positive self-concept and, in turn, enhancing one’s self-esteem^5,6,9^. This dynamic is fundamentally contrastive, whereby the group identified with (the in-group) is positively represented in opposition to those outside the group (the out-group/s). The perceived distinctiveness of the in-group from the out-group is integral to the identity’s subjective utility^6^. When the clarity of the differentiation is degraded, including due to holding competing identities, the utility of identification is diminished^10^.

In recent decades, research exploring the psychological effects of social identification—typically evincing its subjective benefits—has proliferated, often offering accounts heavily informed by SIT persistently (for instance, see:^2,11–14^. This research corpus has exhibited a high degree of diversity in focus, investigating identifications ranging from with one’s school^15,16^, with one’s workplace social groups^14^, to with one’s minority regional group^17^. However, of present interest is the large swath of studies that investigate and, in many cases, evince the positive psychological effects of the most superordinate forms of identification, namely with one’s nation^2,16,18–22^, on the one hand, and the world^16,19,23^, on the other.

What theoretical account best interprets these findings? Given abundant evidence for the positive psychological effects of social identities of a wide variety, perhaps the most straightforward inference from the evidence is that social identification in general is beneficial for psychological well-being. However, upon closer examination, studies on national and global identification often imply more specific, independently plausible explanations of their psychological mechanisms and relationships with well-being. On the one hand, that national identification is linked to well-being largely accords with the characterization of social identity advanced by social identity theory. In identifying with one’s nation, the individual is essentially differentiating herself as part of a national group in contrast to those of other nations^24^, thereby—as per SIT—promoting a positive self-concept and self-esteem^4,25^. This account is often assumed both by those who defend national identity and those who criticize it, with the latter questioning whether its subjective benefits outweigh inferred negative social consequences^4^. And indeed, there is substantial empirical evidence for such negative consequences, such as increased xenophobia^26^, reduced cooperation, and less sensitivity to broader global goals^27^.

On the other hand, theoretical discussions concerning global identification (or similar constructs, like identification with all humanity (IWAH)), including its positive subjective potential for the individual, emphasize precisely its inverse nature to national identification: global identification transcends subordinate identifications and is maximally inclusive^24,28^. Independent of the social identity theory tradition, Maslow^29^ offered early speculations about the nature of a global identification, arguing that belonging to humanity is a high level need reached by self-actualized individuals^30^, in line with the Adlerian^31^ concept of “Gemeinschaftsgefühl”, wherein self-actualizing people “have for human beings in general a deep feeling of identification, sympathy, and affection in spite of the occasional anger, impatience or disgust” (p. 217)^29^. Like the behavioral evidence for national identity’s essentially in-group/out-group differentiating psychological mechanism, so too is there behavioral evidence corroborating this expansive, inclusive nature of global identity. Feelings of oneness with humanity, global citizenship, and belonging to the world are associated with reduced social discounting, and increased impartial beneficence^32^. At a higher order, global consciousness subsumes IWAH and impartial prosocial dispositions under a single construct, which latent profile analysis (LPA) indicates to be highly reliable and temporally stable^33,34^. Experiments analyzing global consciousness indicate that its prosocial dispositions do, in fact, translate to prosocial behavior.

Juxtaposed, the two accounts outlined above, one for national and the other for global identity, present an interesting puzzle. Given that they seem to suggest opposing psychological mechanisms—for the former, in-group/out-group differentiation; for the latter, maximal inclusiveness—what should we expect the effect on psychological well-being to be if both identities are jointly held? Various studies have analyzed both identities independently^16,18,19^, but research has yet to examine their interactions. On this inquiry, perhaps the most relevant finding is—as one might intuit—that national and global identity are negatively correlated^24^. From this and their potentially antithetical underlying mechanisms, a reasonably straightforward intuition is that two identities would mutually temper, if not cancel, each other’s positive effects.

However, this theoretical move may not be so immediately obvious in light of the often complicated nature of nested or multiple social identities, a topic of interest already within the early SIT work^35^ but of particular concern within the social identity literature in more recent decades. In a seminal paper underscoring the relevance of social identity to economics, Akerlof and Kranton^36^ proposed a utility function in which social identity—consisting not just of one but the overall relevant set of an individual’s identifications—serves as an argument. In parallel, work on social identity complexity highlights the highly dynamic, context- and individual-dependent interplay between identifications, concluding that in certain cases more identity complexity—which one might expect from strongly holding both national and global identity—may yield greater affective resilience against negative events^37^. In other cases, like when faced with an external threat to one identity, flexibly shifting to another may prove more beneficial^38^. Later empirical research has corroborated the more nuanced approach of social identity complexity, finding that whether holding multiple identities promotes or diminishes well-being is highly contingent on the degree to which an individual has integrated those identities^39,40^. Given this, while individuals who strongly identify with both their nation and the world may be less common, it is an open question as to whether those that do have favorably or poorly integrated the two identities. In fact, there is evidence that, for those high in concern for others, more local forms of identification may not be differentiational in nature and instead be simply motivated by a need for inclusion—thereby rendering local and global identities compatible^41^.

Thus, we address the identified empirical gap—understanding the joint effects of national and global identity on psychological well-being—by analyzing data on national citizenship identification, national pride, and global citizenship identification, as our primary predictors, and life satisfaction, as our criterion, from the World Values Survey. Inspired by the aforementioned utility function proposed by Akerlof and Kranton^36^, we ground our approach on the following conceptual model:

$$\:\:{U}_{i}=f\left({I}_{i}\right),\text{s}\text{u}\text{c}\text{h}\:\text{t}\text{h}\text{a}\text{t}\:{LS}_{i}\:{\cong\:\:U}_{i}$$

Here, utility *U*_*i*_ and life satisfaction *LS*_*i*_ of an individual *i* are determined by that individual’s social identifications, *I*_*i*_. In line with both SIT and Akerlof and Kranton^36^, we also assume that individuals can simultaneously hold multiple identifications, which they may flexibly activate depending on the situation they find themselves in. In the following, we briefly situate our approach within existing empirical findings.

Using longitudinal data, Khan and colleagues^20^ found that national identification predicts decreased self-reported anxiety and increased self-reported health. Notably, in motivating their measure of national identification, they advocate (echoing Ha and Jang^22^ a conceptual distinction between measures of national identification and national pride, with the former capturing a cognitive dimension and the latter capturing an affective dimension of national identity, broadly construed. Accordingly, since separate measures of national citizenship identification and national pride are both available in Waves 5 and 6 of the WVS, we opted to include both to see if the conceptual distinction yields different results when tested. There is no world/global analogue of national pride in the data. Similarly, Grozdanovska^42^ found positive associations between national identity and both life satisfaction and meaning in life, although no significant relationship with positive affect. In contrast, Bonetto et al.^21^ found a positive association between national identification and happiness with data collected during the COVID-19 pandemic. Morrison and colleagues^43^ investigated the conceptually related measure of national satisfaction (assessed by using the Ladder of Life scale for country ratings), finding it to be a strong predictor of life satisfaction. Similarly, Reeskens and Wright^44^ found a significant positive association between national pride and life satisfaction. Ha and Jang^22^ found national pride—but not national identity—to be positively associated with happiness. We test the potential asymmetry between national identification and pride by including both as predictors in the present research.

While comparatively less abundant, empirical evidence points to a positive link between IWAH or global identification and psychological well-being. Utilizing a mediation model on data from Turkish adults, Çağış and Erol Korkmaz^23^ found IWAH to positively predict subjective well-being (SWB). However, this effect was indirect: IWAH increased satisfaction of basic psychological needs (BPNS)—which includes the sub-dimensions of autonomy, competence, and relatedness—in turn increasing SWB. In contrast, Hodges and Gore^18^ found that identification with community and country each predicts well-being, but not IWAH. The two most relevant works for present purposes, however, are Greenaway et al.^16^ and Cramer and Pawsey^19^, both of which use the World Values Survey (WVS)—also used in this study. Using Wave 6 of the WVS, Greenaway et al.^16^ found that both global and national identification positively predict well-being (which, as a composite, included life satisfaction). Similarly, using Wave 7, Cramer and Pawsey^19^ found that closeness (a different question block) to one’s country and to the world both predicted increased perceived happiness.

Substantial research has justified using subjective well-being measures as meaningful proxies for experienced utility^45,46^. To this end, we treat life satisfaction as an appropriate measure of subjective well-being^47^. Naturally, one would expect a wide array of other demographic factors to significantly shape life satisfaction, which should be accounted for in isolating the effects of social identity. We refer to Table 1 by Frijters and colleagues^48^ for an initial array of potential factors and, based on availability in the World Values Survey, consider education, employment, perceived income level, marital status, and health. Moreover, in line with Morrison, Tay, and Diener^43^, we also consider satisfaction with finances and gross domestic product per capita (GDPPC). Lastly, we consider age, sex^49^, , and, as a highly theoretically relevant construct, political ideology^50^. Furthermore, we include political ideology to control for any second-order effects of political alignment^51,52^.

**Table 1:** Descriptive statistics by wave.

Variable names	Wave 5 (*N* = 39,879)		Wave 6 (*N* = 60,771)		Waves 5 & 6 (*N* = 100,650)	
	Mean	SD	Mean	SD	Mean	SD
Life satisfaction	6.824	2.232	6.845	2.265	6.837	2.252
World citizenship	3.014	0.829	3.024	0.875	3.020	0.857
National citizenship	3.513	0.598	3.512	0.627	3.512	0.616
National pride	2.515	0.691	2.482	0.704	2.495	0.699
Sex	1.498	0.500	1.509	0.500	1.505	0.500
Age	42.060	16.288	41.441	16.345	41.686	16.325
Marital status	2.679	2.161	2.740	2.191	2.716	2.179
Education	5.419	2.435	5.749	2.368	5.618	2.400
Employment	3.364	2.206	3.427	2.159	3.402	2.178
Income	4.867	2.257	4.843	2.101	4.853	2.164
Health status	2.904	0.839	2.915	0.841	2.910	0.840
Financial satisfaction	5.930	2.427	5.899	2.472	5.911	2.454
Political ideology	5.714	2.404	5.680	2.370	5.693	2.383
GDP Per Capita	18,520.1	14,798.4	18,700.4	14,337.3	18,629.0	14,522.0
ln(GDP Per C)	9.392	1.074	9.491	0.903	9.451	0.976

## Method

### Participants

After preprocessing, our final dataset included 100,650 respondents across 69 nations in Wave 5 (W5, 2005–2009)^53^ and Wave 6 (W6, 2010–2016) of the World Values Survey (WVS)^54^. We selected these two waves since they were the only two waves to include questions explicitly related to world citizenship. The WVS employs a combination of stratified random sampling and probability sampling in its data collection. The nature of our dataset is on two levels, with individuals nested within different countries, preventing us from assuming independence across observations. The sample size for each territory in W5 ranged from a minimum of 479 observations in Morocco to a maximum of 2681 in Egypt. In W6, the smallest sample size constituted 150 participants in Morocco, and the largest sample had 3218 participants in India. In Table 1, we present additional descriptive statistics.

### Variables

Our measures of world citizenship and national citizenship stem from the same question block that read: “People have different views about themselves and how they relate to the world. How strongly do you agree or disagree with the following about how you see yourself? // I see myself as a world citizen // I see myself as a citizen of the [e.g., French] nation”; to which participants responded on a scale of 1–4 (1 = Strongly disagree to 4 = Strongly agree). We then included a measure of national pride (0 = not at all proud to 3 = very proud). Demographic variables included sex (0 = male, 1 = female), age (in years), highest level of education attained (1 = ISCED Level 1 to 9 = ISCED Level 9), perceived income scale (1 = lowest to 10 = highest), employment status (1 = full-time, 2 = part-time, 3 = self-employed, 4 = retired/pensioner, 5 = housewife, 6 = student, 7 = unemployed, 8 = other), marital status (1 = married, 2 = living together as married, 3 = divorced, 4 = separated, 5 = widowed, 6 = single), satisfaction with finances (1 = completely dissatisfied to 10 = completely satisfied), health status (1 = very poor to 4 = very good), and political ideology (1 = Left to 10 = Right). At the country-level, we controlled for log GDP per capita (PPP current international $) sourced from IMF and World Bank data. Life satisfaction was assessed on a 10-point scale (1 = completely dissatisfied to 10 = completely satisfied).

### Analysis

We analyzed our data by following the multilevel procedure previously done by Morrison, Tay, and Diener^43^, and Reeskens and Wright^44^. Beyond the hierarchical nature of the data of individuals nested within countries, we anticipated there to be between-country differences in life satisfaction^55^, suggesting that multilevel modeling is appropriate for our analysis^56^. To this end, we computed the intraclass correlation coefficient (ICC) of life satisfaction to determine how much of the variance in life satisfaction can be attributable to between-country differences^56^. We then followed the prescriptions of Enders and Tofighi^57^ to determine the appropriate centering methods for the explanatory variables in our model. Specifically, since our research questions are tied to the associations between identity and life satisfaction, and interaction effects between identity constructs, we opted for centering within clusters for our identity measures. We group-mean centered all level 1 continuous covariates with meaningfully large ICCs, and grand-mean centered all level 2 predictors.

Moreover, we followed the recommendations by Simmons, Nelson and Simonsohn^58^ and conducted our analyses without and with covariates, introduced in blocks in a sequence of four models, to test for robustness. Our four hierarchical models predicted life satisfaction as a function of: (1) identity variables only; (2) identity variables plus demographic covariates identified by Frijters et al.^48^ as influencing life satisfaction (education, employment, perceived income, marital status, and health status); (3) Model 2 plus financial satisfaction and log GDP per capita, following Morrison, Tay, and Diener^43^; and (4) Model 3 plus age, sex, and political ideology^49,50^. Concretely, our modelling procedure was as follows:

#### Model 1

First, we expressed life satisfaction as a function of identity (identification with world citizenship, national citizenship, and national pride) without covariates.

$$\:\:{LS}_{j,k}=f\left({I}_{j,k}\right)$$

Where $\:{LS}_{j,k}$ represents life satisfaction, $\:{I}_{j,k}$ represents a vector of identity variables, and $\:j$ denotes an individual in country $\:k$. Moreover, we treated country as a random effect to account for between-country differences. We lay out our multilevel equations at the individual and country levels, respectively:

$$\:Level\:1:\:{LS}_{j,k}={\beta\:}_{0k}+{\beta\:}_{1k}{X}_{jk}+{r}_{jk}$$

$$\:Level\:2:\:{\beta\:}_{0k}={\gamma\:}_{00}+{\gamma\:}_{01}{G}_{k}+{U}_{0k};\:$$

$$\:{\beta\:}_{1k}={\gamma\:}_{10}+{\gamma\:}_{11}{G}_{k}+{U}_{1k}$$

Where $\:{X}_{jk}$ represents individual-level predictors, $\:\beta\:$ denotes individual-level coefficients, $\:{G}_{k}$ represents country-level predictors, and $\:\gamma\:$ denotes country-level coefficients, while $\:{r}_{jk}$, $\:{U}_{0k}$, $\:{U}_{1k}$ capture the residual terms.

#### Model 2

Second, we explored the relationship between life satisfaction and the identity variables, while controlling for important life satisfaction predictors per Frijters et al. (Table 1)^48^, including the highest level of education attained, employment status, perceived income scale, marital status, and health status.

$$\:\:{LS}_{j,k}=f({I}_{j,k},\:{D}_{j,k})$$

Where $\:{D}_{j,k}$ represents a vector of demographic variables. We opted to center our continuous covariates for this and further models at their grand-mean^59^; c.f^43^.

#### Model 3

Third, we included additional variables considered from Morrison, Tay, and Diener^43^, including satisfaction with finances and the natural logarithm of GDP per capita.

#### Model 4

Lastly, we added age, sex, and political ideology^49,50^.

To probe significant interactions in our most robust model (Model 4), we conducted simple slopes analyses and estimated life satisfaction means for all two-way combinations of the three identity predictors at three levels each: − 1SD (low), mean (standardized at 0), and + 1SD (high). This yielded nine estimated means for each pair of predictors. We identified the highest estimated mean and conducted eight pairwise comparisons between this maximum and each of the other combinations, applying Bonferroni correction (α/8) to control for multiple comparisons. To corroborate these model-based results, we conducted an analogous empirical analysis using the raw data. Each identity predictor was binned into three categories: low (< − 1SD), medium (–1SD to + 1SD), and high ( > + 1SD). We calculated observed life satisfaction means for all nine combinations within each pair of identity predictors, then conducted the same eight Bonferroni-corrected pairwise comparisons between the highest observed mean and the remaining eight combinations.

## Results

### What are the influences of national pride, national citizenship, world citizenship on life satisfaction?

To investigate how national and global identification influence life satisfaction, we first began with an intercept-only model to establish how much variance of life satisfaction is attributable to between-country differences. The ICC of 0.131 indicated that 13.1% of the variance in life satisfaction is due to between-country differences (see notes in Table 2). As a result, we included random intercepts for countries in all subsequent models.

**Table 2:** Hierarchical regression results for life Satisfaction.

Variable	B	95% CI		SE	β	*R*²	ΔR²
		LL	UL				
**Model 1**: No covariates							
Constant	6.67	2.96	10.38	1.89	0	0.036	0.036
National pride	0.36***	0.34	0.38	0.01	0.1		
National citizenship	0.08***	0.06	0.1	0.01	0.02		
World citizenship	0.16***	0.14	0.17	0.01	0.06		
National pride × National citizenship	0.01	– 0.01	0.04	0.01	0		
National pride × World citizenship	– 0.04***	– 0.06	– 0.02	0.01	– 0.01		
National citizenship × World citizenship	– 0.01	– 0.03	0.01	0.01	0		
**Model 2**: Model 1, controlling for education, employment, perceived income, marital status, and health status							
Constant	2.95	– 0.49	6.4	1.76	-0.97	0.177	0.141
National pride	0.3***	0.28	0.32	0.01	0.08		
National citizenship	0.08***	0.06	0.1	0.01	0.02		
World citizenship	0.1***	0.09	0.11	0.01	0.04		
National pride × National citizenship	0.03*	0	0.05	0.01	0		
National pride × World citizenship	– 0.03**	– 0.05	– 0.01	0.01	– 0.01		
National citizenship × World citizenship	0	– 0.02	0.02	0.01	0		
**Model 3**: Model 2, additionally controlling for satisfaction with finances and ln(GDP Per C)							
Constant	0.84***	– 1.74	3.42	1.32	– 0.62	0.311	0.134
National pride	0.23***	0.22	0.25	0.01	0.06		
National citizenship	0.07***	0.05	0.09	0.01	0.02		
World citizenship	0.07	0.06	0.08	0.01	0.03		
National pride × National citizenship	0.03**	0.01	0.05	0.01	0.01		
National pride × World citizenship	– 0.02*	– 0.04	0	0.01	– 0.01		
National citizenship × World citizenship	0	– 0.02	0.02	0.01	0		
**Model 4**: Model 3, additionally controlling for age, sex, and political ideology							
Constant	– 1.71	– 4.67	1.24	1.51	– 0.62	0.315	0.004
National pride	0.22***	0.21	0.24	0.01	0.06		
National citizenship	0.06***	0.04	0.09	0.01	0.02		
World citizenship	0.07***	0.05	0.08	0.01	0.02		
National pride × National citizenship	0.04**	0.01	0.06	0.01	0.01		
National pride × World citizenship	– 0.02*	– 0.05	0	0.01	– 0.01		
National citizenship × World citizenship	0	– 0.02	0.02	0.01	0		

Note. All identity predictors are at the within-country level. B = unstandardized coefficients. β = standardized coefficients. LL/UL = lower/upper limits of the 95% confidence interval. Intercept-only model: ICC = 0.131, *n* = 130,016 individuals, 75 countries. Model 1: ICC = 0.118, *n* = 130,016 individuals, 75 countries. Model 2: ICC = 0.106, *n* = 129,966 individuals, 75 countries. Model 3: ICC = 0.065, *n* = 129,966 individuals, 75 countries. Model 4: ICC = 0.060, *n* = 100,613 individuals, 69 countries. *p < .05, ** *p* < .01, *** *p* < .001.

Our first model examined whether our three identity measures independently predict life satisfaction without any controls (see Model 1 in Table 2, Supplemental Table 1). This baseline Model 1 tests the fundamental premise of whether these identities matter for individual life satisfaction. In particular, Model 1 revealed that national pride, national citizenship, and world citizenship at the individual (within-country) level were each highly significant predictors of life satisfaction (national pride: *B* = 0.36, *t*(130,004) = 36.14, *p* < .001, 95% CI [0.34, 0.38]; national citizenship: *B* = 0.08, *t*(130,004) = 7.35, *p* < .001, 95% CI [0.06, 0.10]; world citizenship: *B* = 0.16, *t*(130,004) = 21.10, *p* < .001, 95% CI [0.14, 0.17]). The interaction term between national pride and world citizenship was highly significant and negative (*B* = − 0.04, *t*(130,004) = − 3.35, *p* < .001, 95% CI [− 0.06, − 0.02]). In other words, national pride and world citizenship appear to moderate the effects of each other on life satisfaction, such that the positive effect of national pride on life satisfaction is weaker among those who strongly identify as world citizens, and conversely, the positive effect of world citizenship is weaker among those with high national pride. No significant interaction was detected between national pride and national citizenship (*B* = 0.01, *t*(130,004) = 1.13, *p* = .257, 95% CI [− 0.01, 0.04]), nor national citizenship and world citizenship (*B* = − 0.01, *t*(130,004) = − 0.74, *p* = .457, 95% CI [− 0.03, 0.01].)

Our second model introduced our first set of controls^48^: education, employment, marital status, health status, and perceived income scale (see Model 2 in Table 2, Supplemental Table 2). Here, we found that each of national pride (*B* = 0.30, *t*(129,928) = 31.74, *p* < .001, 95% CI [0.28, 0.32]), national citizenship (*B* = 0.08, t(129,928) = 7.54, *p* < .001, 95% CI [0.06, 0.10]), and world citizenship (*B* = 0.10, *t*(129,928) = 14.25, *p* < .001, 95% CI [0.09, 0.11]) were still highly significant predictors of life satisfaction. Moreover, our interaction terms indicated that, with these set of covariates, there is a significantly positive interaction effect between national pride and national citizenship (*B* = 0.03, *t*(129,928) = 2.12, *p* = .034, 95% CI [0.00, 0.05]), and a highly significant negative interaction between national pride and world citizenship (*B* = − 0.03, *t*(129,928) = − 2.75, *p* = .006, 95% CI [− 0.05, − 0.01]). We did not find a significant interaction between national citizenship and world citizenship (*B* = 0.00, *t*(129,928) = 0.01, *p* = .996, 95% CI [− 0.02, 0.02]).

In our third step, we added satisfaction with finances and the natural logarithm of GDP per capita (see Model 3 in Table 2, Supplemental Table 3)^43^. At this step, we again replicate the finding that all three identity variables are highly significant predictors of life satisfaction (national pride: *B* = 0.23, *t*(129,925) = 26.69, *p* < .001, 95% CI [0.22, 0.25]; national citizenship: *B* = 0.07, *t*(129,925) = 7.33, *p* < .001, 95% CI [0.05, 0.09]; world citizenship: *B* = 0.07, *t*(129,925) = 10.90, *p* < .001, 95% CI [0.06, 0.08]). Turning to our interaction analyses, we found a highly significant positive interaction between national pride and national citizenship (*B* = 0.03, *t*(129,925) = 2.86, *p* = .004, 95% CI [0.01, 0.05]) and a significant negative interaction between national pride and world citizenship (*B* = − 0.02, *t*(129,925) = − 2.30, *p* = .021, 95% CI [− 0.04, 0.00]). No significant interaction was detected between national citizenship and world citizenship (*B* = − 0.00, *t*(129,925) = − 0.33, *p* = .738, 95% CI [− 0.02, 0.02]).

In the fourth step, we introduced our last set of controls: age, sex, and political ideology (see Model 4 in Table 2, Supplemental Table 4)^49,50^. The main effects of national pride (*B* = 0.22, *t*(100,567) = 22.93, *p* < .001, 95% CI [0.21, 0.24]), national citizenship (*B* = 0.06, *t*(100,567) = 6.08, *p* < .001, 95% CI [0.04, 0.09]) and world citizenship (*B* = 0.07, *t*(100,567) = 9.28, *p* < .001, 95% CI [0.05, 0.08]) demonstrated the same pattern as in the preceding Models 1–3. Here again, we detected a significant positive interaction effect between national pride and national citizenship (*B* = 0.04, *t*(100,567) = 3.03, *p* = .002, 95% CI [0.01, 0.06]), and a significant negative interaction between national pride and world citizenship (*B* = − 0.02, *t*(100,567) = − 2.11, *p* = .035, 95% CI [− 0.05, 0.00]). We did not detect a significant interaction between national citizenship and world citizenship (*B* = 0.00, *t*(100,567) = 0.01, *p* = .989, 95% CI [− 0.02, 0.02]).

How do national pride, national citizenship, and world citizenship moderate each other on life satisfaction?

**Fig. 1:**
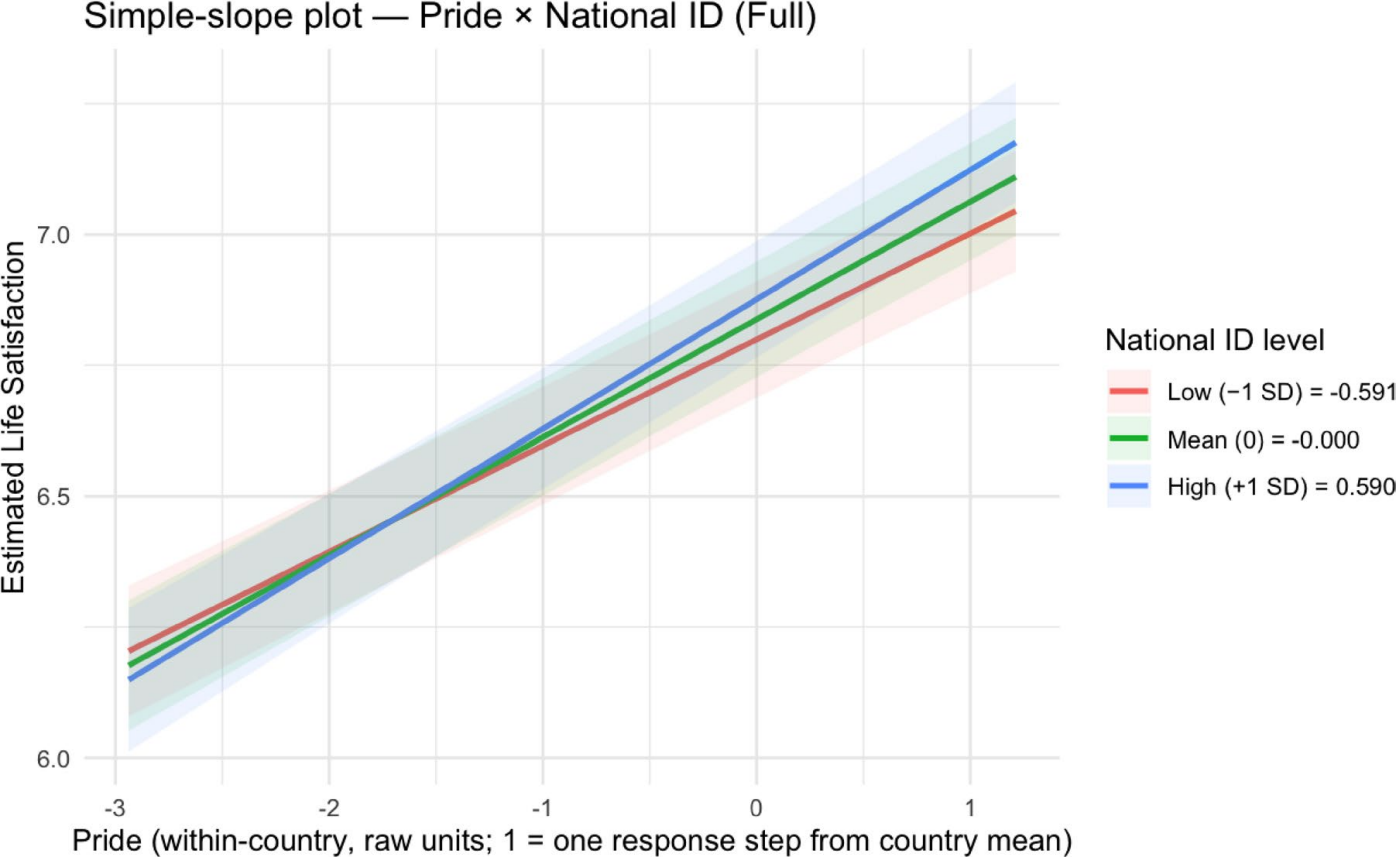
Simple slopes plot for national pride × national citizenship in Model 4.

**Fig. 2:**
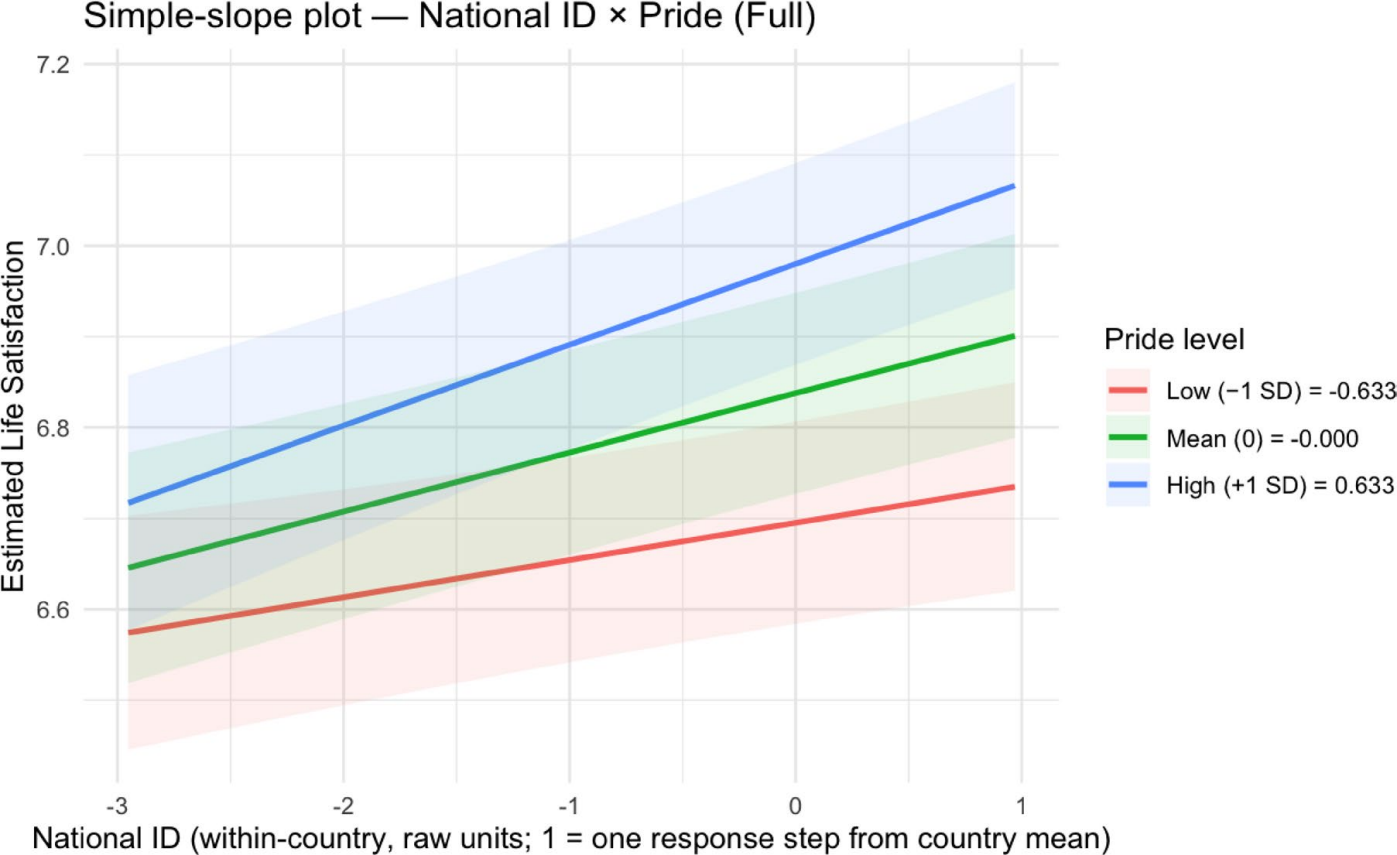
Simple slopes plots for national citizenship × national pride in Model 4.

**Fig. 3:**
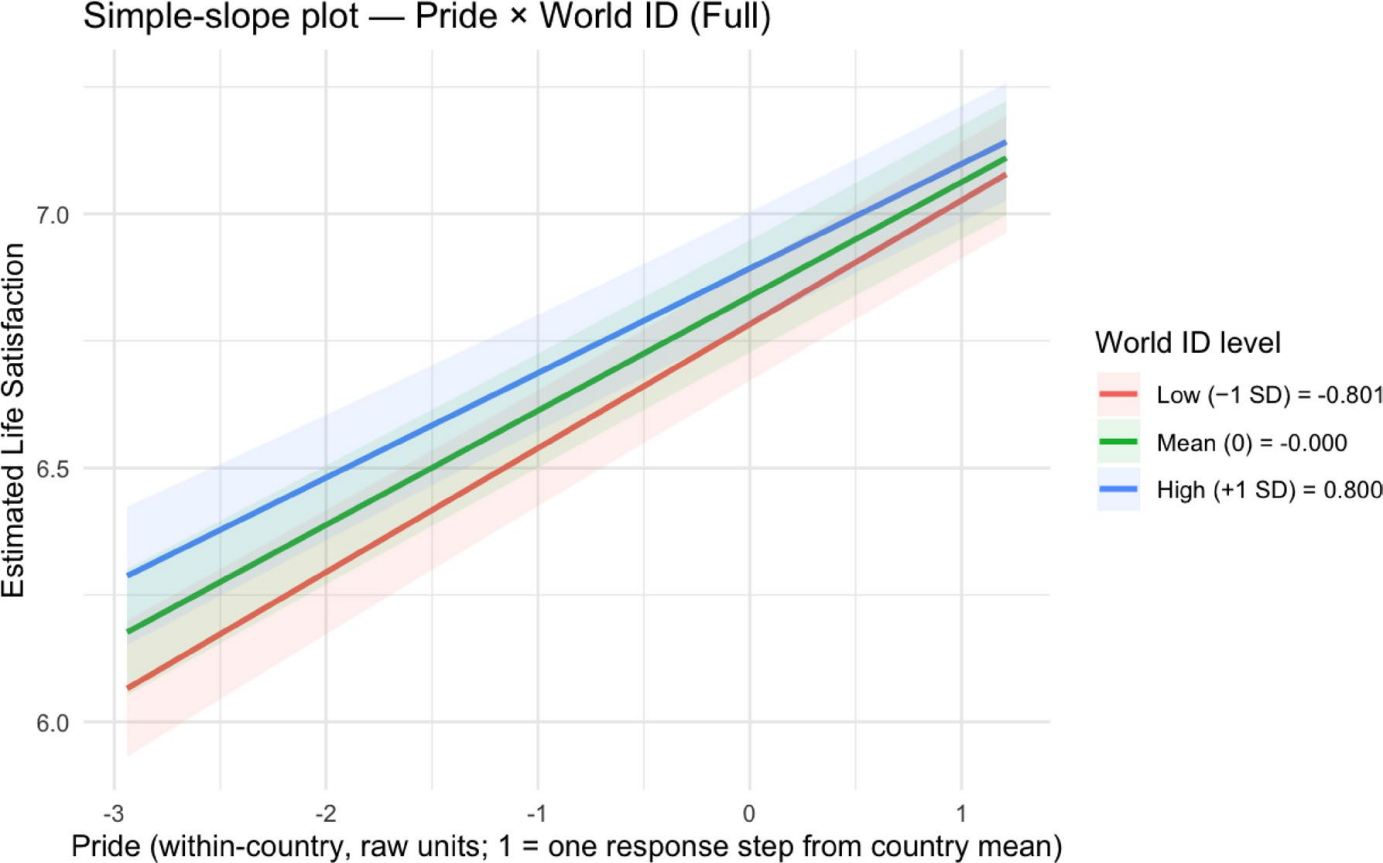
Simple slopes plot for national pride × world citizenship in Model 4.

**Fig. 4:**
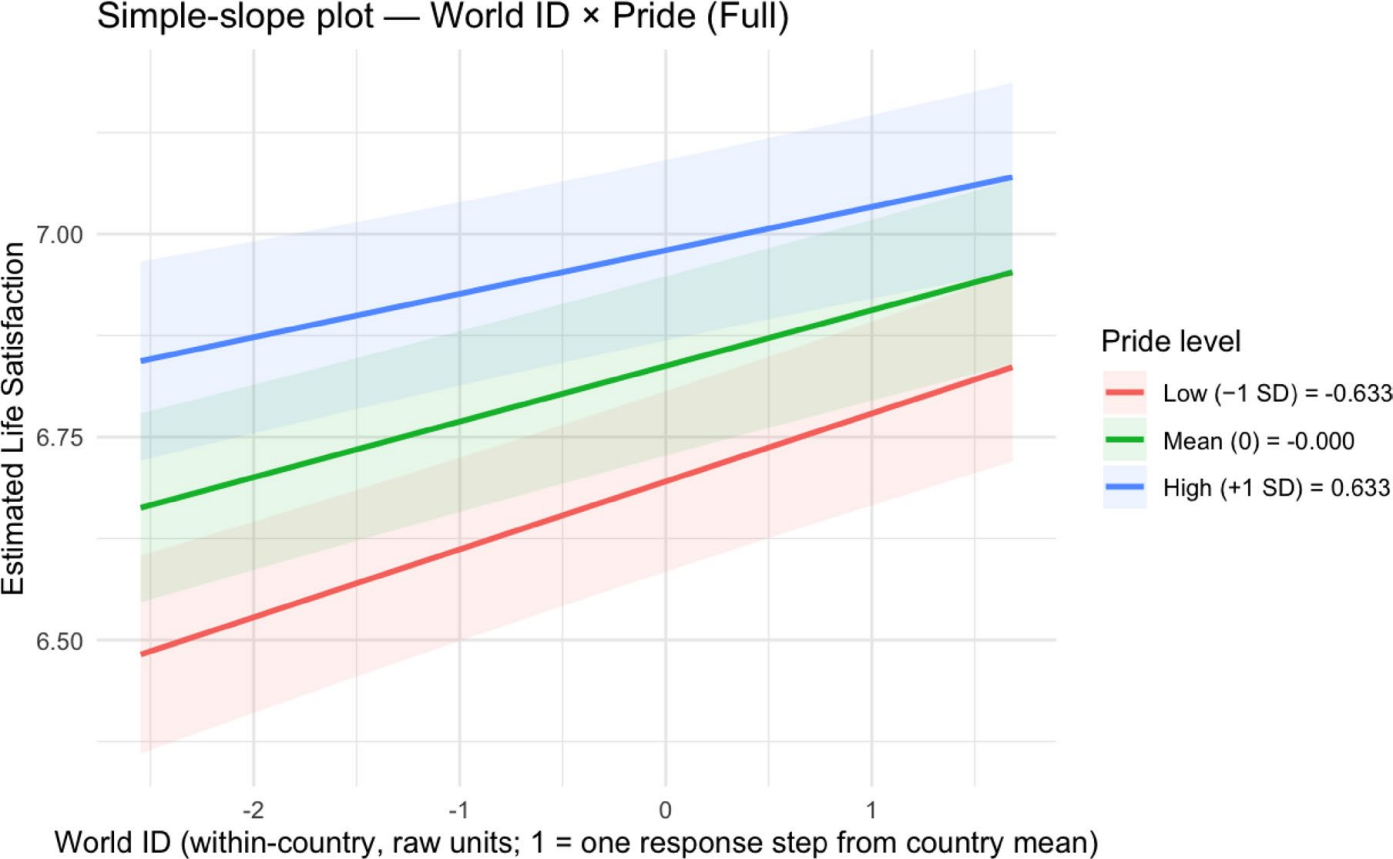
Simple slopes plots for world citizenship × national pride in Model 4.

To understand how national pride, national citizenship, and world citizenship moderate each other’s effects on life satisfaction, we probed the significant Model 4 interactions through simple slopes analyses (see Table 3; Figs. 1, 2, 3 and 4; see Supplemental Tables 5 and Supplemental Figs. 1–2 for non-significant national citizenship × world citizenship interaction).

**Table 3:** Simple slopes analysis for model 4.

National ID level	National ID value	Slope of pride on LS	SE	df	*t*	*p*	Lower 95% CI	Upper 95% CI
Low (− 1 SD)	-0.59	0.20	0.01	100,518.20	18.56	0.000	0.18	0.22
Mean (0)	0.00	0.22	0.01	100,514.80	22.93	0.000	0.21	0.24
High (+ 1 SD)	0.59	0.25	0.01	100,524.30	18.30	0.000	0.22	0.27

Pride level	Pride value	Slope of national ID on LS	SE	df	*t*	*p*	Lower 95% CI	Upper 95% CI
Low (− 1 SD)	-0.63	0.04	0.01	100,516.40	3.38	0.001	0.02	0.06
Mean (0)	0.00	0.06	0.01	100,511.40	6.08	0.000	0.04	0.09
High (+ 1 SD)	0.63	0.09	0.01	100,521.90	6.18	0.000	0.06	0.12

World ID level	World ID value	Slope of pride on LS	SE	df	*t*	*p*	Lower 95% CI	Upper 95% CI
Low (− 1 SD)	-0.80	0.24	0.01	100,520.80	18.70	0.000	0.22	0.27
Mean (0)	0.00	0.22	0.01	100,514.80	22.93	0.000	0.21	0.24
High (+ 1 SD)	0.80	0.21	0.01	100,512.40	15.15	0.000	0.18	0.23

Pride level	Pride value	Slope of World ID on LS	SE	df	*t*	*p*	Lower 95% CI	Upper 95% CI
Low (− 1 SD)	-0.63	0.08	0.01	100,512.60	8.28	0.000	0.06	0.10
Mean (0)	0.00	0.07	0.01	100,512.30	9.28	0.000	0.05	0.08
High (+ 1 SD)	0.63	0.05	0.01	100,517.90	5.14	0.000	0.03	0.07

For the national pride × national citizenship interaction, we found that the positive effect of national pride on life satisfaction varied by level of national citizenship. At low levels of national citizenship (– 1 SD below the mean), the slope of national pride was 0.20 (SE = 0.01, *t*(100,518.20) = 18.56, *p* < .001, 95% CI [0.18, 0.22]). At mean levels of national citizenship, this effect increased to 0.22 (SE = 0.01, *t*(100,514.80) = 22.93, *p* < .001, 95% CI [0.21, 0.24]). At high levels of national citizenship (+ 1 SD above the mean), the effect was strongest at 0.25 (*SE* = 0.01, *t*(100,524.30) = 18.30, *p* < .001, 95% CI [0.22, 0.27]). Conversely, examining national citizenship as the focal predictor, its effect on life satisfaction also increased with national pride levels: *B* = 0.04 (*SE* = 0.01, *t*(100,516.40) = 3.38, *p* = .001, 95% CI [0.02, 0.06]) at low pride (− 1 SD below the mean), *B* = 0.06 (*SE* = 0.01, *t*(100,511.40) = 6.08, *p* < .001, 95% CI [0.04, 0.09]) at mean pride, and *B* = 0.09 (*SE* = 0.01, *t*(100,521.90) = 6.18, *p* < .001, 95% CI [0.06, 0.12]) at high pride (+ 1 SD below the mean).

For the national pride × world citizenship interaction, a different pattern emerged. The positive effect of national pride on life satisfaction was strongest at low levels of world citizenship (− 1 SD below the mean): *B* = 0.24 (*SE* = 0.01, *t*(100,520.80) = 18.70, *p* < .001, 95% CI [0.22, 0.27]). This effect decreased to *B* = 0.22 (*SE* = 0.01, *t*(100,514.80) = 22.93, *p* < .001, 95% CI [0.21, 0.24]) at mean levels of world citizenship and further diminished to *B* = 0.21 (*SE* = 0.01, *t*(100,512.40) = 15.15, *p* < .001, 95% CI [0.18, 0.23]) at high levels of world citizenship (+ 1 SD above the mean). When examining world citizenship as the focal predictor, its positive effect on life satisfaction similarly decreased with increasing national pride: *B* = 0.08 (*SE* = 0.01, *t*(100,512.60) = 8.28, *p* < .001, 95% CI [0.06, 0.10]) at low pride (− 1 SD below the mean), *B* = 0.07 (*SE* = 0.01, *t*(100,512.30) = 9.28, *p* < .001, 95% CI [0.05, 0.08]) at mean pride, and *B* = 0.05 (*SE* = 0.01, *t*(100,517.90) = 5.14, *p* < .001, 95% CI [0.03, 0.07]) at high pride (+ 1 SD above the mean).

### What are the expected consequences of the identity interactions on life satisfaction?

To test the consequence of the interactions on expected life satisfaction at various strengths of the identity variables—with a particular interest in the negative interaction between national pride and world citizenship—we then estimated mean life satisfaction values at + 1SD, − 1SD, and mean of each variable. We summarize these results in Table 4 (national pride × national citizenship), Table 5 (national pride × world citizenship) and Supplemental Table 6.

**Table 4:** Estimated mean life satisfaction from interaction of National pride × National citizenship.

Pride level	Pride value	National ID level	National ID value	Predicted LS	SE	Lower 95% CI	Upper 95% CI
Low (− 1 SD)	– 0.63	Low (− 1 SD)	– 0.59	6.67	0.06	6.56	6.78
Mean (0)	0.00	Low (− 1 SD)	– 0.59	6.80	0.06	6.69	6.91
High (+ 1 SD)	0.63	Low (− 1 SD)	– 0.59	6.93	0.06	6.81	7.04
Low (− 1 SD)	– 0.63	Mean (0)	0.00	6.70	0.06	6.58	6.81
Mean (0)	0.00	Mean (0)	0.00	6.84	0.06	6.73	6.95
High (+ 1 SD)	0.63	Mean (0)	0.00	6.98	0.06	6.87	7.09
Low (− 1 SD)	– 0.63	High (+ 1 SD)	0.59	6.72	0.06	6.61	6.83
Mean (0)	0.00	High (+ 1 SD)	0.59	6.88	0.06	6.77	6.99
High (+ 1 SD)	0.63	High (+ 1 SD)	0.59	7.03	0.06	6.92	7.14

**Table 5:** Estimated mean life satisfaction from interaction of National pride × world citizenship.

Pride level	Pride value	World ID level	World ID value	Predicted LS	SE	Lower 95% CI	Upper 95% CI
Low (− 1 SD)	– 0.63	Low (− 1 SD)	– 0.80	6.63	0.06	6.52	6.74
Mean (0)	0.00	Low (− 1 SD)	– 0.80	6.78	0.06	6.67	6.89
High (+ 1 SD)	0.63	Low (− 1 SD)	– 0.80	6.94	0.06	6.82	7.05
Low (− 1 SD)	– 0.63	Mean (0)	0.00	6.70	0.06	6.58	6.81
Mean (0)	0.00	Mean (0)	0.00	6.84	0.06	6.73	6.95
High (+ 1 SD)	0.63	Mean (0)	0.00	6.98	0.06	6.87	7.09
Low (− 1 SD)	– 0.63	High (+ 1 SD)	0.80	6.76	0.06	6.65	6.87
Mean (0)	0.00	High (+ 1 SD)	0.80	6.89	0.06	6.78	7.00
High (+ 1 SD)	0.63	High (+ 1 SD)	0.80	7.02	0.06	6.91	7.14

**Fig. 5:**
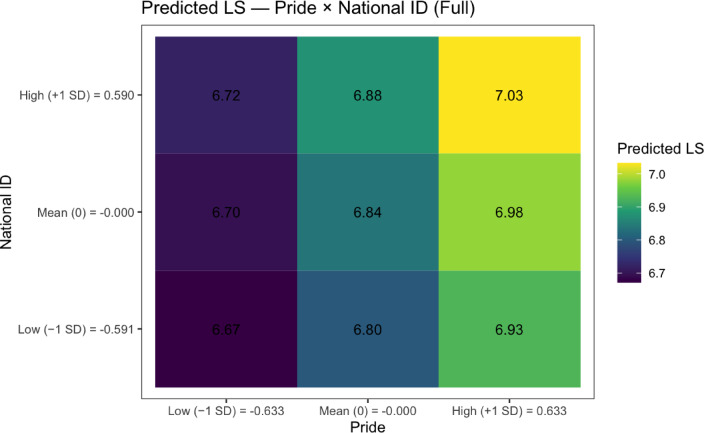
Heat map for Table 4 (national pride × national citizenship)

**Fig. 6:**
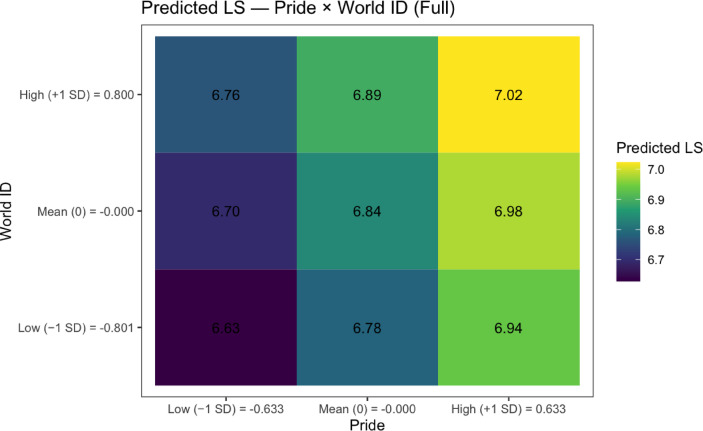
Heat map for Table 5 (national pride × world citizenship)

We found that for national pride × national citizenship, the highest estimated life satisfaction value of 7.03 was yielded by + 1SD national pride and + 1SD national citizenship (*SE* = 0.06, 95% CI [6.92, 7.14]). Similarly, despite the negative interaction, for national pride × world citizenship the highest estimated life satisfaction of 7.02 was yielded by + 1SD national pride and + 1SD world citizenship (*SE* = 0.06, 95% CI [6.91, 7.14]). To test whether these two values were significantly higher than the rest of the estimated life satisfaction values of the different identity strengths, we conducted 8 pair-wise comparisons, the results of which are summarized in Table 6 (see Supplemental Table 7). For the national pride × national citizenship interaction, individuals high on both identities (+ 1 SD above the mean) showed significantly higher life satisfaction than all other combinations (all *p* < .001). The differences ranged from 0.05 (*SE* = 0.01, z = 6.18, 95% CI [0.03, 0.08]) when compared to high pride/mean national citizenship, to 0.36 (*SE* = 0.02, z = 23.11, 95% CI [0.32, 0.40]) when compared to low pride/low national citizenship. Similarly, for the national pride × world citizenship interaction, individuals high on both identities showed significantly higher life satisfaction than all other combinations (all *p* < .001). The differences ranged from 0.04 (*SE* = 0.01, z = 5.14, 95% CI [0.02, 0.07]) when compared to low pride/low world citizenship, to 0.39 (*SE* = 0.02, z = 23.52, 95% CI [0.35, 0.44]) when compared to low pride/low world citizenship. These findings suggest that despite the negative interaction between national pride and world citizenship, individuals who strongly endorse both identities experience the highest levels of life satisfaction (see Figs. 5 and 6, Supplemental Figs. 3–6, and Supplemental Tables 8–9 for our empirical analyses, which corroborate our findings).

**Table 6:** Pairwise comparisons (Bonferroni/8-adjusted) for National pride × National citizenship and National pride × world citizenship.

Contrast	Estimate	SE	df	*z*	Adjusted-*p*	Lower 95% CI	Upper 95% CI
*Pride x National Citizenship*							
(Both High) − (Pride: Low (− 1 SD) = –0.633, National ID: Low (− 1 SD) = –0.591)	0.36	0.02	Inf	23.11	0.000	0.32	0.40
(Both High) − (Pride: Mean (0) = –0.000, National ID: Low (− 1 SD) = –0.591)	0.23	0.01	Inf	15.75	0.000	0.19	0.27
(Both High) − (Pride: High (+ 1 SD) = 0.633, National ID: Low (− 1 SD) = –0.591)	0.11	0.02	Inf	6.18	0.000	0.06	0.15
(Both High) − (Pride: Low (− 1 SD) = –0.633, National ID: Mean (0) = –0.000)	0.34	0.01	Inf	22.87	0.000	0.30	0.38
(Both High) − (Pride: Mean (0) = –0.000, National ID: Mean (0) = –0.000)	0.19	0.01	Inf	18.90	0.000	0.17	0.22
(Both High) − (Pride: High (+ 1 SD) = 0.633, National ID: Mean (0) = –0.000)	0.05	0.01	Inf	6.18	0.000	0.03	0.08
(Both High) − (Pride: Low (− 1 SD) = –0.633, National ID: High (+ 1 SD) = 0.590)	0.31	0.02	Inf	18.30	0.000	0.27	0.36
(Both High) − (Pride: Mean (0) = –0.000, National ID: High (+ 1 SD) = 0.590)	0.16	0.01	Inf	18.30	0.000	0.13	0.18
*Pride x World Citizenship*							
(Both High) − (Pride: Low (− 1 SD) = –0.633, World ID: Low (− 1 SD) = –0.801)	0.39	0.02	Inf	23.52	0.000	0.35	0.44
(Both High) − (Pride: Mean (0) = –0.000, World ID: Low (− 1 SD) = –0.801)	0.24	0.01	Inf	16.50	0.000	0.20	0.28
(Both High) − (Pride: High (+ 1 SD) = 0.633, World ID: Low (− 1 SD) = –0.801)	0.09	0.02	Inf	5.14	0.000	0.04	0.13
(Both High) − (Pride: Low (− 1 SD) = –0.633, World ID: Mean (0) = –0.000)	0.33	0.01	Inf	21.93	0.000	0.29	0.37
(Both High) − (Pride: Mean (0) = –0.000, World ID: Mean (0) = –0.000)	0.19	0.01	Inf	17.83	0.000	0.16	0.21
(Both High) − (Pride: High (+ 1 SD) = 0.633, World ID: Mean (0) = –0.000)	0.04	0.01	Inf	5.14	0.000	0.02	0.07
(Both High) − (Pride: Low (− 1 SD) = –0.633, World ID: High (+ 1 SD) = 0.800)	0.26	0.02	Inf	15.15	0.000	0.21	0.31
(Both High) − (Pride: Mean (0) = –0.000, World ID: High (+ 1 SD) = 0.800)	0.13	0.01	Inf	15.15	0.000	0.11	0.15

## Discussion

We set out to explore how identification with national and global citizenship, as well as national pride, shape life satisfaction. In a series of four models of increasing robustness, we consistently found that each significantly predicted life satisfaction. Analyzed holistically, that both our world identity variables and our two national identity variables were associated with life satisfaction lends credence to the idea that social identification in general, regardless of who (since, on a reasonable view, these global and national identities are radically distinct), is beneficial for well-being. At the same time, upon inspecting the results of our interaction analyses, we found that national pride and world citizenship had a negative interaction in Models 1, 2, and 4, suggesting that—in line with implied contrasting mechanisms—the two identities interfere with each other’s links to well-being. In other words, at first glance, it may seem that maximizing one’s well-being requires choosing one identity or the other. This inference from their negative interaction is falsified by our simple slopes probing on Model 4, where we find that—despite potentially decreasing marginal returns in life satisfaction on one identity as the level of the other identity is increased—the identities are still largely additive in effect. Our estimated means analyses revealed, as expected, that the highest life satisfaction was still yielded on our model from feeling strong national pride and strongly identifying with the world. We then tested this claim for, and found, significance. Moreover, we ran additional empirical, ‘model-free’ analyses, closely paralleling the procedure of our model-based analysis, and found that the group that felt strong national pride and strongly identified with the world had the highest mean life satisfaction.

Additionally, national pride and national citizenship identification had a positive significant interaction from Model 2 through Model 4, which is less surprising. As such, our evidence suggests that their combined effects on life satisfaction are not merely additive but synergistic. Our model-based estimated means comparisons results corroborated this insight, with strongly identifying with one’s nation and feeling strong national pride yielding a statistically significantly higher mean life satisfaction than all other combinations of strengths between the two identities. Similarly, our model-free, empirical analyses reproduced this conclusion.

Given the cross-sectional nature of our analysis, we cannot determine causality, and we cannot rule out reverse causal relationships. For instance, one could argue that happier people are by nature more inclusive, and have stronger feelings of global citizenship. The present set-up prevents us from offering answers to these claims, but we hope to open the door to new avenues of research in this direction. For instance, our findings could be used to develop an intervention that motivates people to adopt a more expansive social identity by highlighting its benefits for their well-being and life satisfaction. Another potential follow-up we envision would be to experimentally manipulate an educational intervention that teaches people about how having more expansive and inclusive group identities can contribute to their life satisfaction. Moreover, additional research would be warranted in the effects of such a manipulation on different outcome measures, such as, but not limited to, the identification with all of humanity scale^60^, donations to charities that maximize welfare to humanity^32^, donations to charities that help people in one’s country of origin, and/or other elements of subjective well-being (e.g., affective, eudaimonic). We are also curious to know how people learn to increase their sense of belonging to broader groups, and how this learning mechanism could influence their circles of moral concern.

In summary, in this work we found further evidence, focusing on the two superordinate identifications of one’s nation and with the world, corroborating the hypothesis that social identification in general is important for life satisfaction. However, more pointedly, we find evidence against the intuition that national and world identification essentially work in contrast in their relation to well-being. While they may mutually temper each other’s positive effects, ultimately their contributions to well-being remain additive. This is so even after controlling for an array of variables for which there is strong evidence to suspect they may influence life satisfaction. In other words, while potentially less straightforward as choosing one or the other, it may be the most beneficial to identify simultaneously with both your nation and the world; cosmopolitan patriots may be better off, surprisingly, than both cosmopolitans and patriots. Framed another way, that those who identify strongly with their nation may gain additional life satisfaction from identifying with the world may serve as a strong motivator to enlarge one’s circle of identification. As mentioned, there is strong evidence of positive behavioral consequences associated with identifying with all of humanity, and it may not be that these consequences really require sacrificing one’s sense of their national in-group.

## Supplementary Information

Below is the link to the electronic supplementary material.

Supplementary Material 1

## Data Availability

Publicly available datasets were used for this study. This data can be downloaded from https://www.worldvaluessurvey.org/WVSContents.jsp. Moreover, we make our analysis scripts and data used from the WVS available on our Open Science Framework repository page here: https://osf.io/cs6gq/.
