# Supplementary material for: Life satisfaction effects of national identity, global identity, and their interactions
Source: Sci Rep. 2025 Dec 8;16:66. doi: 10.1038/s41598-025-29471-8 (PMC12764469; doi:10.1038/s41598-025-29471-8)
Supplement: Supplementary file 1 — Supplementary Material 1 [file 41598_2025_29471_MOESM1_ESM.docx]

| **Supplemental Figure 1.** Simple slopes plots for national citizenship × world citizenship in Model 4.  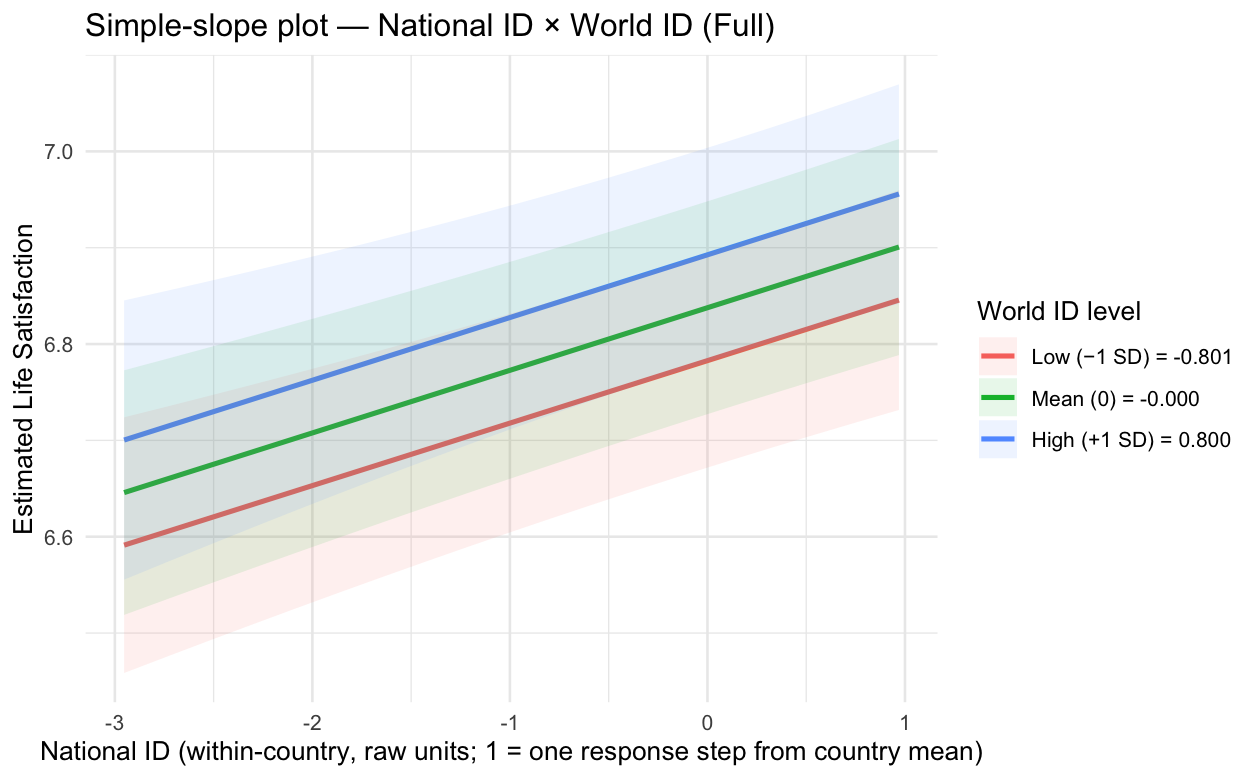  **Supplemental Figure 2.** Simple slopes plots for world citizenship x national citizenship in Model 4.  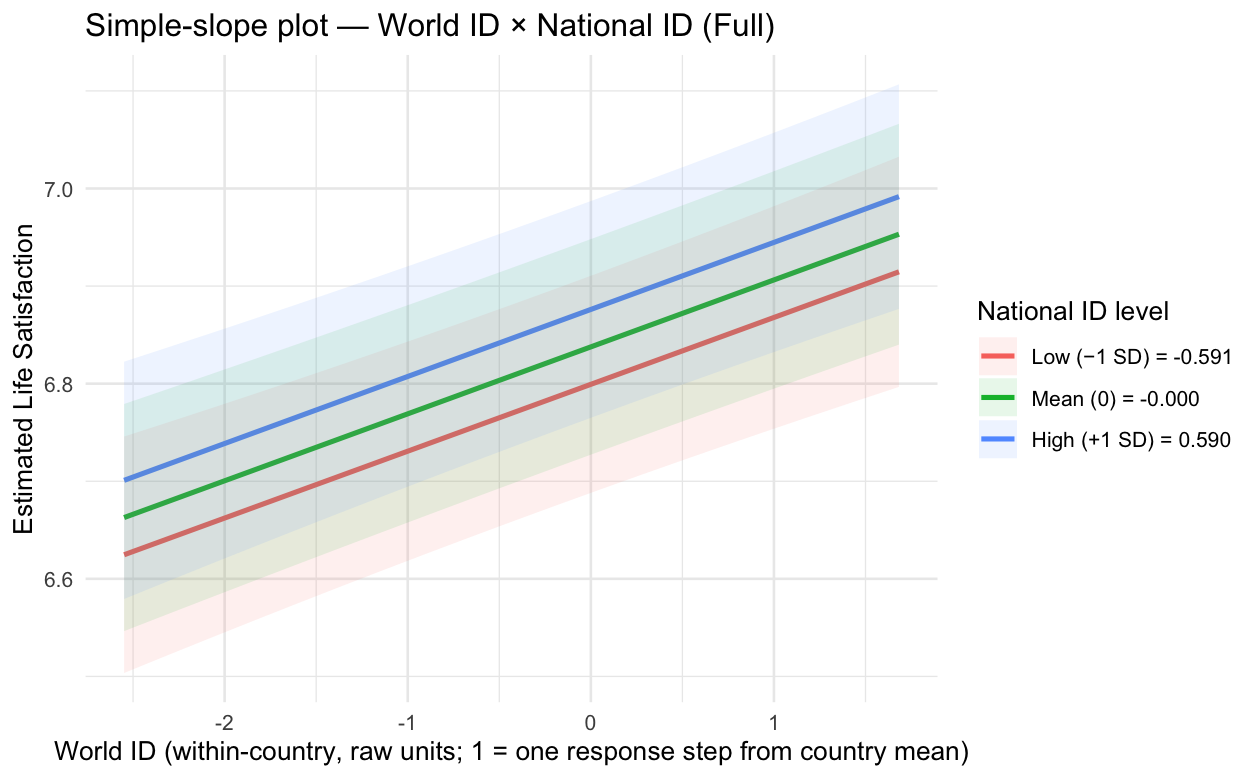 |
| --- |

|  |
| --- |

**Supplemental Figure 3.** Heat map for national citizenship × world citizenship


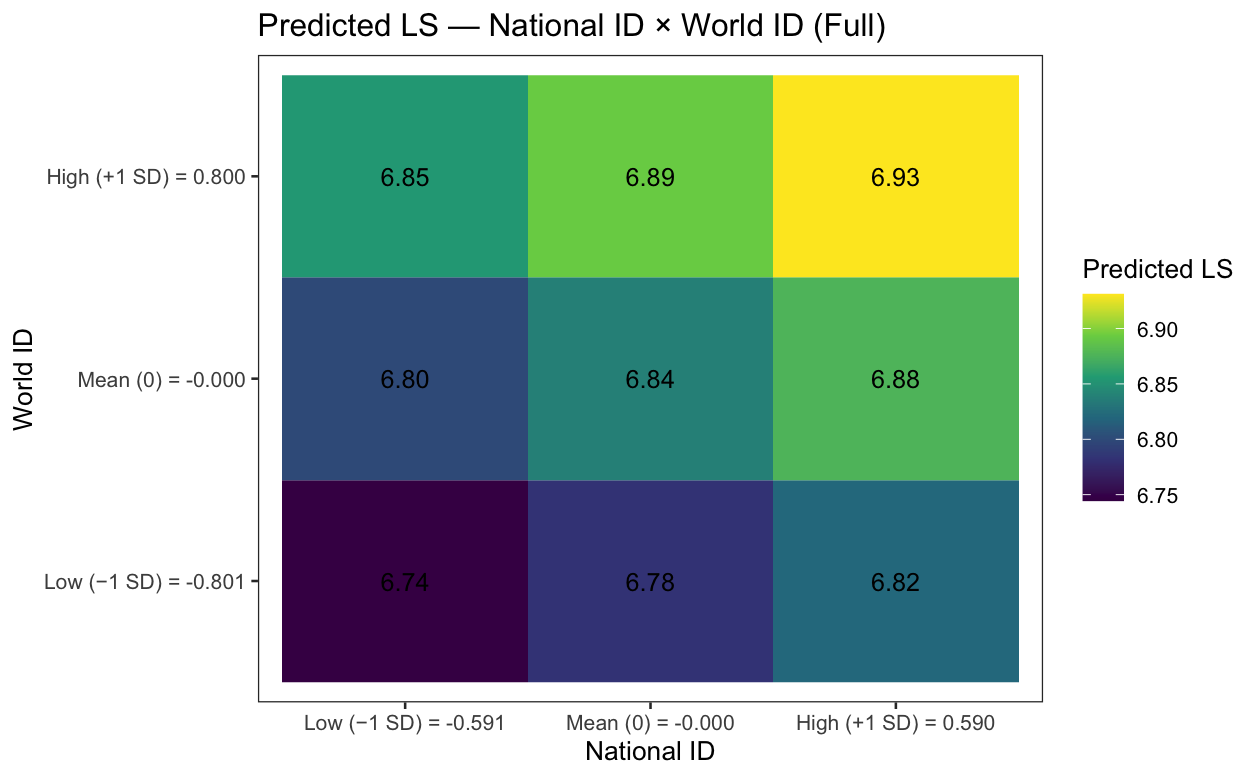


**Supplemental Figure 4.** Empirical heat map for national pride × national citizenship


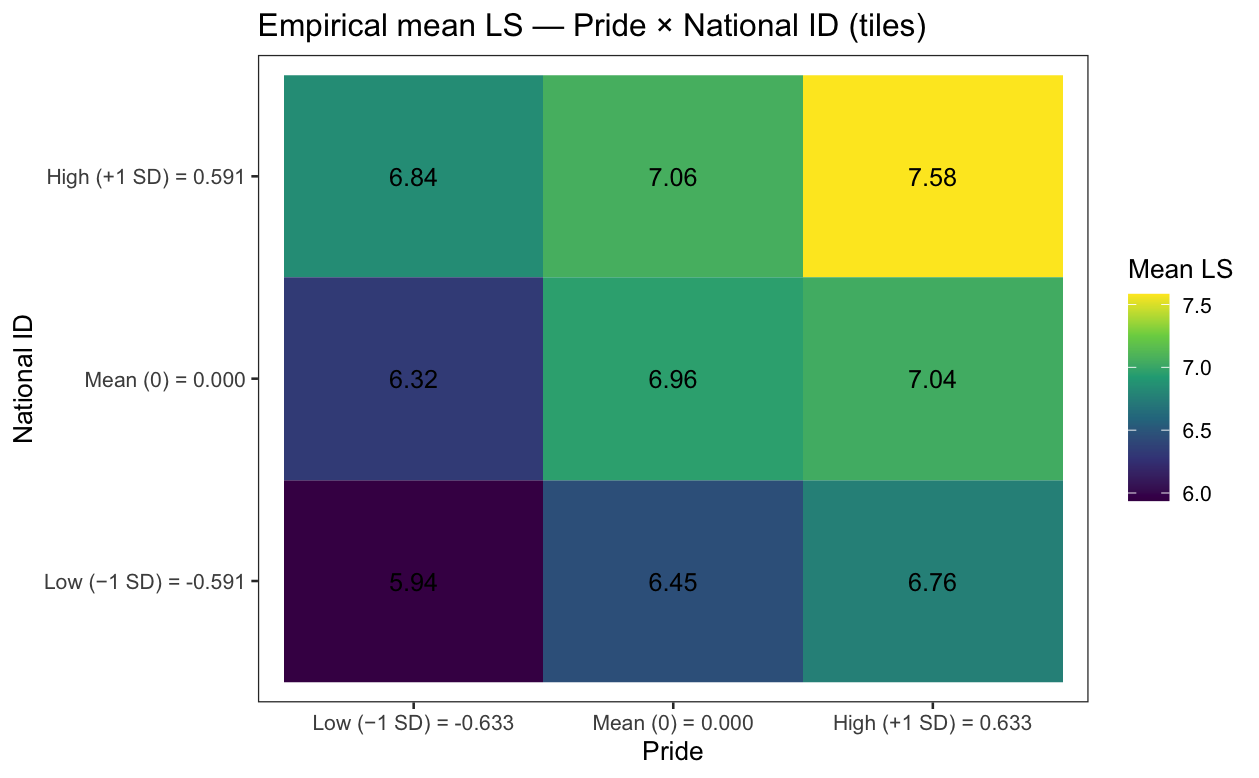


**Supplemental Figure 5.** Empirical heat map for national pride × world citizenship


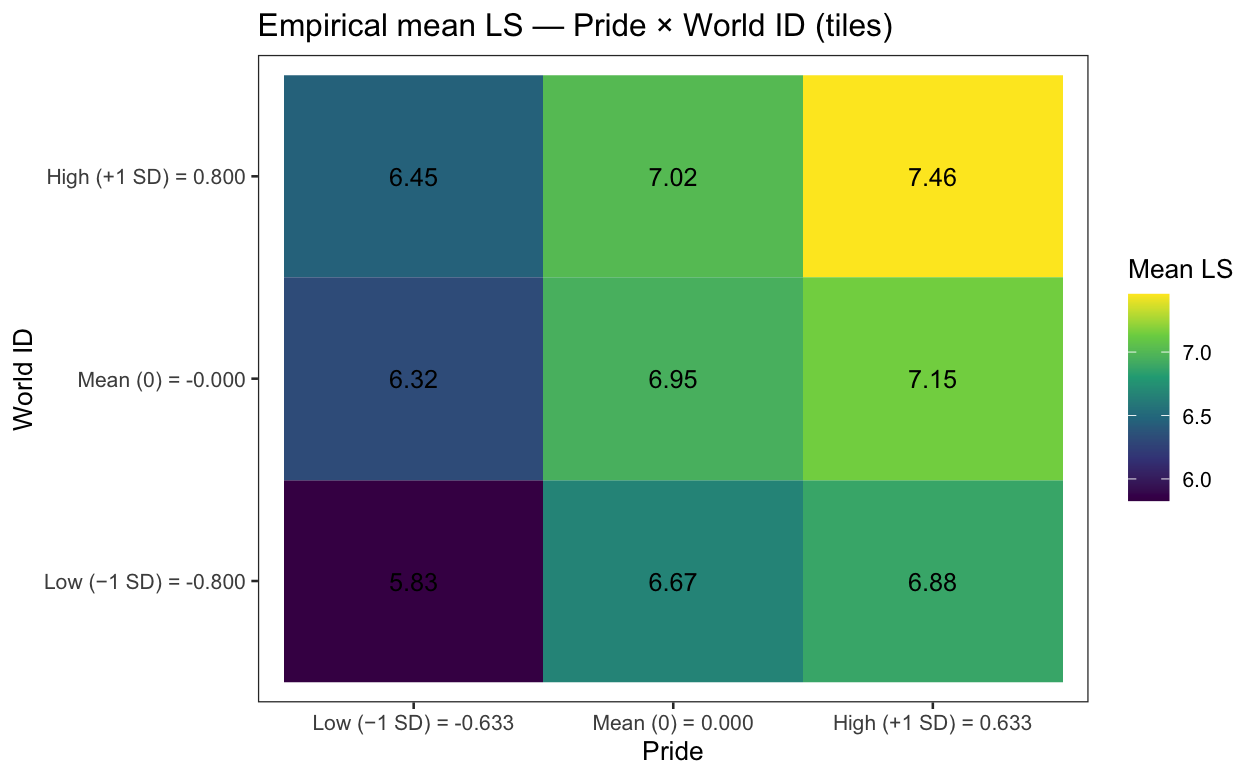


**Supplemental Figure 6.** Empirical heat map for national citizenship × world citizenship


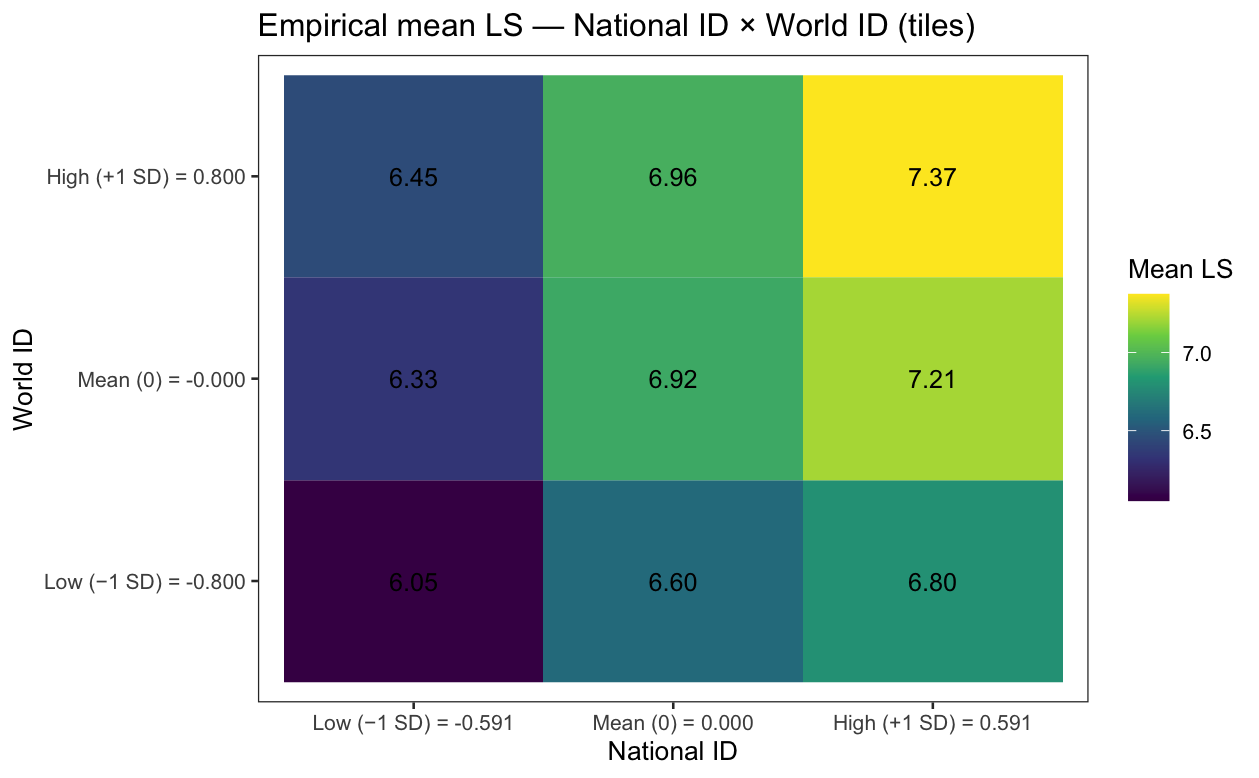


**Supplemental Table 1.** Regression Results of Identity Predictors (Model 1) on Life Satisfaction

| **Variable** | **B** | **LL** | **UL** | **SE** | **β** | **t (df)** | **p** |
| --- | --- | --- | --- | --- | --- | --- | --- |
| (Intercept) | 6.67 | 2.96 | 10.38 | 1.89 | 0 | 3.52 (130004) | < .001 |
| pride_within | 0.36 | 0.34 | 0.38 | 0.01 | 0.1 | 36.14 (130004) | < .001 |
| nation_within | 0.08 | 0.06 | 0.1 | 0.01 | 0.02 | 7.35 (130004) | < .001 |
| world_within | 0.16 | 0.14 | 0.17 | 0.01 | 0.06 | 21.1 (130004) | < .001 |
| pride_between | 0.26 | -0.57 | 1.1 | 0.42 | 0.03 | 0.62 (130004) | 0.533 |
| nation_between | -0.95 | -2.15 | 0.26 | 0.61 | -0.07 | -1.54 (130004) | 0.123 |
| world_between | 0.93 | 0.28 | 1.57 | 0.33 | 0.12 | 2.82 (130004) | 0.005 |
| pride_within:nation_within | 0.01 | -0.01 | 0.04 | 0.01 | 0 | 1.13 (130004) | 0.257 |
| pride_within:world_within | -0.04 | -0.06 | -0.02 | 0.01 | -0.01 | -3.35 (130004) | <.001 |
| nation_within:world_within | -0.01 | -0.03 | 0.01 | 0.01 | 0 | -0.74 (130004) | 0.457 |

R² (marginal) = 0.036 | R² (conditional) = 0.150 | ICC = 0.118 | n = 130016 individuals, 75 countries

**Supplemental Table 2.** Regression Results of Identity Predictors with First Set of Covariates (Model 2) on Life Satisfaction

| **Variable** | **B** | **LL** | **UL** | **SE** | **β** | **t (df)** | **p** |
| --- | --- | --- | --- | --- | --- | --- | --- |
| (Intercept) | 2.95 | -0.49 | 6.4 | 1.76 | -0.97 | 1.68 (129928) | 0.093 |
| pride_within | 0.3 | 0.28 | 0.32 | 0.01 | 0.08 | 31.74 (129928) | < .001 |
| nation_within | 0.08 | 0.06 | 0.1 | 0.01 | 0.02 | 7.54 (129928) | < .001 |
| world_within | 0.1 | 0.09 | 0.11 | 0.01 | 0.04 | 14.25 (129928) | < .001 |
| pride_between | 0.39 | -0.39 | 1.17 | 0.4 | 0.05 | 0.98 (129928) | 0.327 |
| nation_between | -0.79 | -1.85 | 0.27 | 0.54 | -0.06 | -1.46 (129928) | 0.144 |
| world_between | 0.74 | 0.18 | 1.3 | 0.29 | 0.10 | 2.57 (129928) | 0.01 |
| d_edu_within | 0 | 0 | 0.01 | 0.00 | 0.00 | 1.56 (129928) | 0.12 |
| d_edu_between | 0.22 | 0.06 | 0.38 | 0.08 | 0.10 | 2.64 (129928) | 0.01 |
| d_emp2 | -0.02 | -0.06 | 0.02 | 0.02 | -0.01 | -0.85 (129928) | 0.40 |
| d_emp3 | -0.04 | -0.08 | 0 | 0.02 | -0.02 | -2.16 (129928) | 0.03 |
| d_emp4 | 0.25 | 0.21 | 0.29 | 0.02 | 0.11 | 12.11 (129928) | < .001 |
| d_emp5 | 0.17 | 0.13 | 0.2 | 0.02 | 0.07 | 8.8 (129928) | < .001 |
| d_emp6 | 0.22 | 0.17 | 0.27 | 0.02 | 0.1 | 9.01 (129928) | < .001 |
| d_emp7 | -0.24 | -0.28 | -0.19 | 0.02 | -0.1 | -11.24 (129928) | < .001 |
| d_emp8 | 0.04 | -0.04 | 0.12 | 0.04 | 0.02 | 0.88 (129928) | 0.381 |
| d_mar2 | -0.13 | -0.18 | -0.09 | 0.02 | -0.06 | -5.73 (129928) | < .001 |
| d_mar3 | -0.4 | -0.46 | -0.35 | 0.03 | -0.18 | -13.44 (129928) | < .001 |
| d_mar4 | -0.45 | -0.53 | -0.37 | 0.04 | -0.2 | -10.8 (129928) | < .001 |
| d_mar5 | -0.16 | -0.21 | -0.11 | 0.02 | -0.07 | -6.48 (129928) | < .001 |
| d_mar6 | -0.24 | -0.27 | -0.21 | 0.02 | -0.11 | -15.69 (129928) | < .001 |
| d_hea2 | 0.98 | 0.93 | 1.03 | 0.03 | 0.43 | 38.61 (129928) | < .001 |
| d_hea3 | 1.63 | 1.58 | 1.68 | 0.02 | 0.71 | 65.3 (129928) | < .001 |
| d_hea4 | 2.22 | 2.17 | 2.27 | 0.03 | 0.97 | 83.88 (129928) | < .001 |
| d_inc2 | 0.15 | 0.1 | 0.2 | 0.03 | 0.07 | 5.48 (129928) | < .001 |
| d_inc3 | 0.28 | 0.23 | 0.33 | 0.03 | 0.12 | 11.29 (129928) | < .001 |
| d_inc4 | 0.54 | 0.49 | 0.59 | 0.02 | 0.24 | 21.93 (129928) | < .001 |
| d_inc5 | 0.78 | 0.74 | 0.83 | 0.02 | 0.34 | 33.36 (129928) | < .001 |
| d_inc6 | 0.99 | 0.95 | 1.04 | 0.03 | 0.44 | 39.7 (129928) | < .001 |
| d_inc7 | 1.24 | 1.19 | 1.3 | 0.03 | 0.55 | 47.28 (129928) | < .001 |
| d_inc8 | 1.45 | 1.39 | 1.51 | 0.03 | 0.63 | 48.77 (129928) | < .001 |
| d_inc9 | 1.48 | 1.39 | 1.56 | 0.04 | 0.65 | 34.29 (129928) | < .001 |
| d_inc10 | 1.56 | 1.47 | 1.65 | 0.05 | 0.68 | 34.45 (129928) | < .001 |
| pride_within:nation_within | 0.03 | 0 | 0.05 | 0.01 | 0 | 2.12 (129928) | 0.034 |
| pride_within:world_within | -0.03 | -0.05 | -0.01 | 0.01 | -0.01 | -2.75 (129928) | 0.006 |
| nation_within:world_within | 0 | -0.02 | 0.02 | 0.01 | 0 | 0 (129928) | 0.996 |

R² (marginal) = 0.177 | R² (conditional) = 0.264 | ICC = 0.106 | n = 129966 individuals, 75 countries

**Supplemental Table 3.** Regression Results of Identity Predictors with Second Set of Covariates (Model 3) on Life Satisfaction

| **Variable** | **B** | **LL** | **UL** | **SE** | **β** | **t (df)** | **p** |
| --- | --- | --- | --- | --- | --- | --- | --- |
| (Intercept) | 0.84 | -1.74 | 3.42 | 1.32 | -0.62 | 0.64 (129925) | 0.525 |
| pride_within | 0.23 | 0.22 | 0.25 | 0.01 | 0.06 | 26.69 (129925) | < .001 |
| nation_within | 0.07 | 0.05 | 0.09 | 0.01 | 0.02 | 7.33 (129925) | < .001 |
| world_within | 0.07 | 0.06 | 0.08 | 0.01 | 0.03 | 10.9 (129925) | < .001 |
| pride_between | 0.28 | -0.29 | 0.84 | 0.29 | 0.04 | 0.97 (129925) | 0.334 |
| nation_between | 0.06 | -0.73 | 0.85 | 0.4 | 0 | 0.15 (129925) | 0.883 |
| world_between | 0.38 | -0.06 | 0.83 | 0.23 | 0.05 | 1.68 (129925) | 0.093 |
| d_edu_within | 0 | -0.01 | 0 | 0 | 0 | -1.25 (129925) | 0.212 |
| d_edu_between | 0.02 | -0.11 | 0.14 | 0.06 | 0.01 | 0.3 (129925) | 0.763 |
| d_emp2 | 0 | -0.04 | 0.04 | 0.02 | 0 | -0.19 (129925) | 0.852 |
| d_emp3 | -0.04 | -0.08 | -0.01 | 0.02 | -0.02 | -2.3 (129925) | 0.022 |
| d_emp4 | 0.09 | 0.06 | 0.13 | 0.02 | 0.04 | 4.99 (129925) | < .001 |
| d_emp5 | 0.13 | 0.1 | 0.17 | 0.02 | 0.06 | 7.41 (129925) | < .001 |
| d_emp6 | 0.18 | 0.14 | 0.23 | 0.02 | 0.08 | 7.99 (129925) | < .001 |
| d_emp7 | -0.13 | -0.17 | -0.09 | 0.02 | -0.06 | -6.72 (129925) | < .001 |
| d_emp8 | 0.04 | -0.03 | 0.12 | 0.04 | 0.02 | 1.11 (129925) | 0.265 |
| d_mar2 | -0.08 | -0.13 | -0.04 | 0.02 | -0.04 | -3.86 (129925) | < .001 |
| d_mar3 | -0.29 | -0.34 | -0.23 | 0.03 | -0.13 | -10.2 (129925) | < .001 |
| d_mar4 | -0.29 | -0.37 | -0.21 | 0.04 | -0.13 | -7.46 (129925) | < .001 |
| d_mar5 | -0.13 | -0.18 | -0.09 | 0.02 | -0.06 | -5.79 (129925) | < .001 |
| d_mar6 | -0.2 | -0.23 | -0.17 | 0.01 | -0.09 | -14.01 (129925) | < .001 |
| d_hea2 | 0.76 | 0.71 | 0.81 | 0.02 | 0.33 | 32.09 (129925) | < .001 |
| d_hea3 | 1.26 | 1.21 | 1.3 | 0.02 | 0.55 | 53.74 (129925) | < .001 |
| d_hea4 | 1.72 | 1.68 | 1.77 | 0.02 | 0.76 | 69.08 (129925) | < .001 |
| d_inc2 | 0.06 | 0.01 | 0.11 | 0.03 | 0.03 | 2.39 (129925) | 0.017 |
| d_inc3 | 0.07 | 0.02 | 0.11 | 0.02 | 0.03 | 2.84 (129925) | 0.004 |
| d_inc4 | 0.18 | 0.13 | 0.23 | 0.02 | 0.08 | 7.76 (129925) | < .001 |
| d_inc5 | 0.28 | 0.24 | 0.33 | 0.02 | 0.12 | 12.74 (129925) | < .001 |
| d_inc6 | 0.35 | 0.3 | 0.4 | 0.02 | 0.15 | 14.64 (129925) | < .001 |
| d_inc7 | 0.47 | 0.42 | 0.52 | 0.03 | 0.21 | 18.62 (129925) | < .001 |
| d_inc8 | 0.55 | 0.5 | 0.61 | 0.03 | 0.24 | 19.46 (129925) | < .001 |
| d_inc9 | 0.57 | 0.49 | 0.65 | 0.04 | 0.25 | 13.91 (129925) | < .001 |
| d_inc10 | 0.61 | 0.53 | 0.69 | 0.04 | 0.27 | 14.25 (129925) | < .001 |
| d_fin_within | 0.33 | 0.33 | 0.34 | 0 | 0.34 | 138.62 (129925) | < .001 |
| d_fin_between | 0.41 | 0.26 | 0.56 | 0.08 | 0.15 | 5.31 (129925) | < .001 |
| log_GDP_c | 0.25 | 0.17 | 0.33 | 0.04 | 0.11 | 6.43 (129925) | < .001 |
| pride_within:nation_within | 0.03 | 0.01 | 0.05 | 0.01 | 0.01 | 2.86 (129925) | 0.004 |
| pride_within:world_within | -0.02 | -0.04 | 0 | 0.01 | -0.01 | -2.3 (129925) | 0.021 |
| nation_within:world_within | 0 | -0.02 | 0.02 | 0.01 | 0 | -0.33 (129925) | 0.738 |

R² (marginal) = 0.311 | R² (conditional) = 0.356 | ICC = 0.065 | n = 129966 individuals, 75 countries

**Supplemental Table 4.** Regression Results of Identity Predictors with Final/Full Set of Covariates (Model 4) on Life Satisfaction

| **Variable** | **B** | **LL** | **UL** | **SE** | **β** | **t (df)** | **p** |
| --- | --- | --- | --- | --- | --- | --- | --- |
| (Intercept) | -1.71 | -4.67 | 1.24 | 1.51 | -0.62 | -1.14 (100567) | 0.256 |
| pride_within | 0.22 | 0.21 | 0.24 | 0.01 | 0.06 | 22.93 (100567) | < .001 |
| nation_within | 0.06 | 0.04 | 0.09 | 0.01 | 0.02 | 6.08 (100567) | < .001 |
| world_within | 0.07 | 0.05 | 0.08 | 0.01 | 0.02 | 9.28 (100567) | < .001 |
| pride_between | 0.62 | 0.02 | 1.21 | 0.3 | 0.08 | 2.03 (100567) | 0.043 |
| nation_between | 0.05 | -0.76 | 0.85 | 0.41 | 0 | 0.12 (100567) | 0.905 |
| world_between | 0.46 | 0 | 0.92 | 0.23 | 0.06 | 1.95 (100567) | 0.051 |
| d_pol_within | 0.03 | 0.02 | 0.03 | 0 | 0.03 | 10.94 (100567) | < .001 |
| d_pol_between | 0.02 | -0.14 | 0.18 | 0.08 | 0.01 | 0.24 (100567) | 0.81 |
| d_fin_within | 0.33 | 0.32 | 0.33 | 0 | 0.34 | 120.78 (100567) | < .001 |
| d_fin_between | 0.4 | 0.24 | 0.57 | 0.08 | 0.15 | 4.83 (100567) | < .001 |
| d_age_within | 0 | 0 | 0 | 0 | 0 | 1.26 (100567) | 0.207 |
| d_age_between | 0.03 | 0.01 | 0.06 | 0.01 | 0.08 | 2.34 (100567) | 0.019 |
| d_edu_within | 0 | -0.01 | 0 | 0 | 0 | -1.08 (100567) | 0.281 |
| d_edu_between | 0.04 | -0.1 | 0.18 | 0.07 | 0.02 | 0.59 (100567) | 0.554 |
| log_GDP_c | 0.19 | 0.1 | 0.28 | 0.05 | 0.08 | 3.99 (100567) | < .001 |
| d_hea2 | 0.74 | 0.68 | 0.79 | 0.03 | 0.33 | 26.91 (100567) | < .001 |
| d_hea3 | 1.24 | 1.19 | 1.3 | 0.03 | 0.55 | 45.94 (100567) | < .001 |
| d_hea4 | 1.7 | 1.65 | 1.76 | 0.03 | 0.76 | 58.9 (100567) | < .001 |
| d_sex2 | 0.1 | 0.07 | 0.12 | 0.01 | 0.04 | 7.57 (100567) | < .001 |
| d_emp2 | -0.01 | -0.06 | 0.03 | 0.02 | 0 | -0.47 (100567) | 0.635 |
| d_emp3 | -0.04 | -0.08 | 0 | 0.02 | -0.02 | -2.06 (100567) | 0.04 |
| d_emp4 | 0.09 | 0.04 | 0.13 | 0.02 | 0.04 | 3.74 (100567) | < .001 |
| d_emp5 | 0.06 | 0.01 | 0.1 | 0.02 | 0.03 | 2.61 (100567) | 0.009 |
| d_emp6 | 0.18 | 0.13 | 0.24 | 0.03 | 0.08 | 6.96 (100567) | < .001 |
| d_emp7 | -0.13 | -0.17 | -0.08 | 0.02 | -0.06 | -5.8 (100567) | < .001 |
| d_emp8 | 0 | -0.08 | 0.08 | 0.04 | 0 | -0.06 (100567) | 0.955 |
| d_mar2 | -0.07 | -0.12 | -0.02 | 0.02 | -0.03 | -2.94 (100567) | 0.003 |
| d_mar3 | -0.31 | -0.37 | -0.25 | 0.03 | -0.14 | -10.16 (100567) | < .001 |
| d_mar4 | -0.29 | -0.37 | -0.2 | 0.04 | -0.13 | -6.72 (100567) | < .001 |
| d_mar5 | -0.2 | -0.25 | -0.15 | 0.03 | -0.09 | -7.4 (100567) | < .001 |
| d_mar6 | -0.17 | -0.2 | -0.13 | 0.02 | -0.07 | -9.67 (100567) | < .001 |
| d_inc2 | 0.05 | -0.01 | 0.11 | 0.03 | 0.02 | 1.74 (100567) | 0.082 |
| d_inc3 | 0.04 | -0.01 | 0.09 | 0.03 | 0.02 | 1.43 (100567) | 0.153 |
| d_inc4 | 0.15 | 0.1 | 0.2 | 0.03 | 0.07 | 5.84 (100567) | < .001 |
| d_inc5 | 0.26 | 0.21 | 0.3 | 0.03 | 0.11 | 10.12 (100567) | < .001 |
| d_inc6 | 0.32 | 0.27 | 0.37 | 0.03 | 0.14 | 11.9 (100567) | < .001 |
| d_inc7 | 0.43 | 0.37 | 0.48 | 0.03 | 0.19 | 15.09 (100567) | < .001 |
| d_inc8 | 0.54 | 0.47 | 0.6 | 0.03 | 0.24 | 16.83 (100567) | < .001 |
| d_inc9 | 0.54 | 0.46 | 0.63 | 0.05 | 0.24 | 12.08 (100567) | < .001 |
| d_inc10 | 0.56 | 0.47 | 0.65 | 0.05 | 0.25 | 11.94 (100567) | < .001 |
| pride_within:nation_within | 0.04 | 0.01 | 0.06 | 0.01 | 0.01 | 3.03 (100567) | 0.002 |
| pride_within:world_within | -0.02 | -0.05 | 0 | 0.01 | -0.01 | -2.11 (100567) | 0.035 |
| nation_within:world_within | 0 | -0.02 | 0.02 | 0.01 | 0 | 0.01 (100567) | 0.989 |

R² (marginal) = 0.315 | R² (conditional) = 0.356 | ICC = 0.060 | n = 100613 individuals, 69 countries

**Supplemental Table 5.** Complete simple slopes analysis for Model 4

| **National ID Level** | **National ID value** | **Slope of Pride on LS** | **SE** | **df** | **t** | **p** | **Lower 95% CI** | **Upper 95% CI** |
| --- | --- | --- | --- | --- | --- | --- | --- | --- |
| Low (−1 SD) | -0.59 | 0.20 | 0.01 | 100,518.20 | 18.56 | 0.000 | 0.18 | 0.22 |
| Mean (0) | 0.00 | 0.22 | 0.01 | 100,514.80 | 22.93 | 0.000 | 0.21 | 0.24 |
| High (+1 SD) | 0.59 | 0.25 | 0.01 | 100,524.30 | 18.30 | 0.000 | 0.22 | 0.27 |
| **Pride Level** | **Pride value** | **Slope of National ID on LS** | **SE** | **df** | **t** | **p** | **Lower 95% CI** | **Upper 95% CI** |
| Low (−1 SD) | -0.63 | 0.04 | 0.01 | 100,516.40 | 3.38 | 0.001 | 0.02 | 0.06 |
| Mean (0) | 0.00 | 0.06 | 0.01 | 100,511.40 | 6.08 | 0.000 | 0.04 | 0.09 |
| High (+1 SD) | 0.63 | 0.09 | 0.01 | 100,521.90 | 6.18 | 0.000 | 0.06 | 0.12 |
| **World ID Level** | **World ID value** | **Slope of Pride on LS** | **SE** | **df** | **t** | **p** | **Lower 95% CI** | **Upper 95% CI** |
| Low (−1 SD) | -0.80 | 0.24 | 0.01 | 100,520.80 | 18.70 | 0.000 | 0.22 | 0.27 |
| Mean (0) | 0.00 | 0.22 | 0.01 | 100,514.80 | 22.93 | 0.000 | 0.21 | 0.24 |
| High (+1 SD) | 0.80 | 0.21 | 0.01 | 100,512.40 | 15.15 | 0.000 | 0.18 | 0.23 |
| **Pride Level** | **Pride value** | **Slope of World ID on LS** | **SE** | **df** | **t** | **p** | **Lower 95% CI** | **Upper 95% CI** |
| Low (−1 SD) | -0.63 | 0.08 | 0.01 | 100,512.60 | 8.28 | 0.000 | 0.06 | 0.10 |
| Mean (0) | 0.00 | 0.07 | 0.01 | 100,512.30 | 9.28 | 0.000 | 0.05 | 0.08 |
| High (+1 SD) | 0.63 | 0.05 | 0.01 | 100,517.90 | 5.14 | 0.000 | 0.03 | 0.07 |
| **World ID Level** | **World ID value** | **Slope of National ID on LS** | **SE** | **df** | **t** | **p** | **Lower 95% CI** | **Upper 95% CI** |
| Low (−1 SD) | -0.80 | 0.06 | 0.01 | 100,521.70 | 5.03 | 0.000 | 0.04 | 0.09 |
| Mean (0) | 0.00 | 0.06 | 0.01 | 100,511.40 | 6.08 | 0.000 | 0.04 | 0.09 |
| High (+1 SD) | 0.80 | 0.07 | 0.02 | 100,514.50 | 4.23 | 0.000 | 0.03 | 0.10 |
| **National ID Level** | **National ID value** | **Slope of World ID on LS** | **SE** | **df** | **t** | **p** | **Lower 95% CI** | **Upper 95% CI** |
| Low (−1 SD) | -0.59 | 0.07 | 0.01 | 100,513.60 | 6.47 | 0.000 | 0.05 | 0.09 |
| Mean (0) | 0.00 | 0.07 | 0.01 | 100,512.30 | 9.28 | 0.000 | 0.05 | 0.08 |
| High (+1 SD) | 0.59 | 0.07 | 0.01 | 100,524.00 | 7.15 | 0.000 | 0.05 | 0.09 |

**Supplemental Table 6.** Estimated mean life satisfaction from each interaction for Model 4

| **Pride Level** | **Pride value** | **National ID Level** | **National ID value** | **Predicted LS** | **SE** | **Lower 95% CI** | **Upper 95% CI** |
| --- | --- | --- | --- | --- | --- | --- | --- |
| Low (−1 SD) | -0.63 | Low (−1 SD) | -0.59 | 6.67 | 0.06 | 6.56 | 6.78 |
| Mean (0) | 0.00 | Low (−1 SD) | -0.59 | 6.80 | 0.06 | 6.69 | 6.91 |
| High (+1 SD) | 0.63 | Low (−1 SD) | -0.59 | 6.93 | 0.06 | 6.81 | 7.04 |
| Low (−1 SD) | -0.63 | Mean (0) | 0.00 | 6.70 | 0.06 | 6.58 | 6.81 |
| Mean (0) | 0.00 | Mean (0) | 0.00 | 6.84 | 0.06 | 6.73 | 6.95 |
| High (+1 SD) | 0.63 | Mean (0) | 0.00 | 6.98 | 0.06 | 6.87 | 7.09 |
| Low (−1 SD) | -0.63 | High (+1 SD) | 0.59 | 6.72 | 0.06 | 6.61 | 6.83 |
| Mean (0) | 0.00 | High (+1 SD) | 0.59 | 6.88 | 0.06 | 6.77 | 6.99 |
| High (+1 SD) | 0.63 | High (+1 SD) | 0.59 | 7.03 | 0.06 | 6.92 | 7.14 |
| **Pride Level** | **Pride value** | **World ID Level** | **World ID value** | **Predicted LS** | **SE** | **Lower 95% CI** | **Upper 95% CI** |
| Low (−1 SD) | -0.63 | Low (−1 SD) | -0.80 | 6.63 | 0.06 | 6.52 | 6.74 |
| Mean (0) | 0.00 | Low (−1 SD) | -0.80 | 6.78 | 0.06 | 6.67 | 6.89 |
| High (+1 SD) | 0.63 | Low (−1 SD) | -0.80 | 6.94 | 0.06 | 6.82 | 7.05 |
| Low (−1 SD) | -0.63 | Mean (0) | 0.00 | 6.70 | 0.06 | 6.58 | 6.81 |
| Mean (0) | 0.00 | Mean (0) | 0.00 | 6.84 | 0.06 | 6.73 | 6.95 |
| High (+1 SD) | 0.63 | Mean (0) | 0.00 | 6.98 | 0.06 | 6.87 | 7.09 |
| Low (−1 SD) | -0.63 | High (+1 SD) | 0.80 | 6.76 | 0.06 | 6.65 | 6.87 |
| Mean (0) | 0.00 | High (+1 SD) | 0.80 | 6.89 | 0.06 | 6.78 | 7.00 |
| High (+1 SD) | 0.63 | High (+1 SD) | 0.80 | 7.02 | 0.06 | 6.91 | 7.14 |
| **National ID Level** | **National ID value** | **World ID Level** | **World ID value** | **Predicted LS** | **SE** | **Lower 95% CI** | **Upper 95% CI** |
| Low (−1 SD) | -0.59 | Low (−1 SD) | -0.80 | 6.74 | 0.06 | 6.63 | 6.86 |
| Mean (0) | 0.00 | Low (−1 SD) | -0.80 | 6.78 | 0.06 | 6.67 | 6.89 |
| High (+1 SD) | 0.59 | Low (−1 SD) | -0.80 | 6.82 | 0.06 | 6.71 | 6.93 |
| Low (−1 SD) | -0.59 | Mean (0) | 0.00 | 6.80 | 0.06 | 6.69 | 6.91 |
| Mean (0) | 0.00 | Mean (0) | 0.00 | 6.84 | 0.06 | 6.73 | 6.95 |
| High (+1 SD) | 0.59 | Mean (0) | 0.00 | 6.88 | 0.06 | 6.77 | 6.99 |
| Low (−1 SD) | -0.59 | High (+1 SD) | 0.80 | 6.85 | 0.06 | 6.74 | 6.97 |
| Mean (0) | 0.00 | High (+1 SD) | 0.80 | 6.89 | 0.06 | 6.78 | 7.00 |
| High (+1 SD) | 0.59 | High (+1 SD) | 0.80 | 6.93 | 0.06 | 6.82 | 7.04 |

**Supplemental Table 7.** Pairwise comparisons (Bonferroni/8-adjusted) for each interaction for Model 4

| **Contrast** | **Estimate** | **SE** | **df** | **z** | **adjusted-*p*** | **Lower 95% CI** | **Upper 95% CI** |
| --- | --- | --- | --- | --- | --- | --- | --- |
| **Pride x National Citizenship** |  |  |  |  |  |  |  |
| (Both High) − (Pride: Low (−1 SD) = -0.633, National ID: Low (−1 SD) = -0.591) | 0.36 | 0.02 | Inf | 23.11 | < .001 | 0.32 | 0.40 |
| (Both High) − (Pride: Mean (0) = -0.000, National ID: Low (−1 SD) = -0.591) | 0.23 | 0.01 | Inf | 15.75 | < .001 | 0.19 | 0.27 |
| (Both High) − (Pride: High (+1 SD) = 0.633, National ID: Low (−1 SD) = -0.591) | 0.11 | 0.02 | Inf | 6.18 | < .001 | 0.06 | 0.15 |
| (Both High) − (Pride: Low (−1 SD) = -0.633, National ID: Mean (0) = -0.000) | 0.34 | 0.01 | Inf | 22.87 | < .001 | 0.30 | 0.38 |
| (Both High) − (Pride: Mean (0) = -0.000, National ID: Mean (0) = -0.000) | 0.19 | 0.01 | Inf | 18.90 | < .001 | 0.17 | 0.22 |
| (Both High) − (Pride: High (+1 SD) = 0.633, National ID: Mean (0) = -0.000) | 0.05 | 0.01 | Inf | 6.18 | < .001 | 0.03 | 0.08 |
| (Both High) − (Pride: Low (−1 SD) = -0.633, National ID: High (+1 SD) = 0.590) | 0.31 | 0.02 | Inf | 18.30 | < .001 | 0.27 | 0.36 |
| (Both High) − (Pride: Mean (0) = -0.000, National ID: High (+1 SD) = 0.590) | 0.16 | 0.01 | Inf | 18.30 | < .001 | 0.13 | 0.18 |
| **Pride x World Citizenship** |  |  |  |  |  |  |  |
| (Both High) − (Pride: Low (−1 SD) = -0.633, World ID: Low (−1 SD) = -0.801) | 0.39 | 0.02 | Inf | 23.52 | < .001 | 0.35 | 0.44 |
| (Both High) − (Pride: Mean (0) = -0.000, World ID: Low (−1 SD) = -0.801) | 0.24 | 0.01 | Inf | 16.50 | < .001 | 0.20 | 0.28 |
| (Both High) − (Pride: High (+1 SD) = 0.633, World ID: Low (−1 SD) = -0.801) | 0.09 | 0.02 | Inf | 5.14 | < .001 | 0.04 | 0.13 |
| (Both High) − (Pride: Low (−1 SD) = -0.633, World ID: Mean (0) = -0.000) | 0.33 | 0.01 | Inf | 21.93 | < .001 | 0.29 | 0.37 |
| (Both High) − (Pride: Mean (0) = -0.000, World ID: Mean (0) = -0.000) | 0.19 | 0.01 | Inf | 17.83 | < .001 | 0.16 | 0.21 |
| (Both High) − (Pride: High (+1 SD) = 0.633, World ID: Mean (0) = -0.000) | 0.04 | 0.01 | Inf | 5.14 | < .001 | 0.02 | 0.07 |
| (Both High) − (Pride: Low (−1 SD) = -0.633, World ID: High (+1 SD) = 0.800) | 0.26 | 0.02 | Inf | 15.15 | < .001 | 0.21 | 0.31 |
| (Both High) − (Pride: Mean (0) = -0.000, World ID: High (+1 SD) = 0.800) | 0.13 | 0.01 | Inf | 15.15 | < .001 | 0.11 | 0.15 |
| **National Citizenship x World Citizenship** |  |  |  |  |  |  |  |
| (Both High) − (National ID: Low (−1 SD) = -0.591, World ID: Low (−1 SD) = -0.801) | 0.19 | 0.02 | Inf | 12.16 | < .001 | 0.14 | 0.23 |
| (Both High) − (National ID: Mean (0) = -0.000, World ID: Low (−1 SD) = -0.801) | 0.15 | 0.01 | Inf | 11.12 | < .001 | 0.11 | 0.18 |
| (Both High) − (National ID: High (+1 SD) = 0.590, World ID: Low (−1 SD) = -0.801) | 0.11 | 0.02 | Inf | 7.15 | < .001 | 0.07 | 0.15 |
| (Both High) − (National ID: Low (−1 SD) = -0.591, World ID: Mean (0) = -0.000) | 0.13 | 0.01 | Inf | 9.06 | < .001 | 0.09 | 0.17 |
| (Both High) − (National ID: Mean (0) = -0.000, World ID: Mean (0) = -0.000) | 0.09 | 0.01 | Inf | 9.56 | < .001 | 0.07 | 0.12 |
| (Both High) − (National ID: High (+1 SD) = 0.590, World ID: Mean (0) = -0.000) | 0.05 | 0.01 | Inf | 7.15 | < .001 | 0.03 | 0.08 |
| (Both High) − (National ID: Low (−1 SD) = -0.591, World ID: High (+1 SD) = 0.800) | 0.08 | 0.02 | Inf | 4.23 | < .001 | 0.03 | 0.13 |
| (Both High) − (National ID: Mean (0) = -0.000, World ID: High (+1 SD) = 0.800) | 0.04 | 0.01 | Inf | 4.23 | < .001 | 0.01 | 0.06 |

Note: adjusted-*p* refers to Bonferroni-corrected for 8 tests (α/8).

**Supplemental Table 8.** Empirical analysis of mean life satisfaction for combinations of identity variables evaluated at low, medium, and high

| **Pair** | **Group** | **n** | **mean** | **SD** | **SE** | **Lower 95% CI** | **Upper 95% CI** |
| --- | --- | --- | --- | --- | --- | --- | --- |
| National ID × World ID (Empirical) | Low_Low | 2,757.00 | 6.05 | 2.42 | 0.05 | 5.96 | 6.14 |
| National ID × World ID (Empirical) | Low_Mid | 8,070.00 | 6.33 | 2.26 | 0.03 | 6.28 | 6.38 |
| National ID × World ID (Empirical) | Low_High | 927.00 | 6.45 | 2.41 | 0.08 | 6.30 | 6.61 |
| National ID × World ID (Empirical) | Mid_Low | 11,860.00 | 6.60 | 2.30 | 0.02 | 6.56 | 6.64 |
| National ID × World ID (Empirical) | Mid_Mid | 57,877.00 | 6.92 | 2.21 | 0.01 | 6.90 | 6.94 |
| National ID × World ID (Empirical) | Mid_High | 10,791.00 | 6.96 | 2.32 | 0.02 | 6.91 | 7.00 |
| National ID × World ID (Empirical) | High_Low | 1,389.00 | 6.80 | 2.30 | 0.06 | 6.68 | 6.92 |
| National ID × World ID (Empirical) | High_Mid | 3,640.00 | 7.21 | 2.00 | 0.03 | 7.15 | 7.28 |
| National ID × World ID (Empirical) | High_High | 3,339.00 | 7.37 | 2.24 | 0.04 | 7.30 | 7.45 |
| Pride × National ID (Empirical) | Low_Low | 4,234.00 | 5.94 | 2.37 | 0.04 | 5.87 | 6.01 |
| Pride × National ID (Empirical) | Low_Mid | 10,387.00 | 6.32 | 2.31 | 0.02 | 6.27 | 6.36 |
| Pride × National ID (Empirical) | Low_High | 664.00 | 6.84 | 2.25 | 0.09 | 6.67 | 7.01 |
| Pride × National ID (Empirical) | Mid_Low | 7,168.00 | 6.45 | 2.26 | 0.03 | 6.40 | 6.50 |
| Pride × National ID (Empirical) | Mid_Mid | 64,957.00 | 6.96 | 2.22 | 0.01 | 6.94 | 6.97 |
| Pride × National ID (Empirical) | Mid_High | 5,076.00 | 7.06 | 2.14 | 0.03 | 7.00 | 7.12 |
| Pride × National ID (Empirical) | High_Low | 352.00 | 6.76 | 2.36 | 0.13 | 6.51 | 7.01 |
| Pride × National ID (Empirical) | High_Mid | 5,184.00 | 7.04 | 2.12 | 0.03 | 6.98 | 7.10 |
| Pride × National ID (Empirical) | High_High | 2,628.00 | 7.58 | 2.12 | 0.04 | 7.50 | 7.66 |
| Pride × World ID (Empirical) | Low_Low | 3,128.00 | 5.83 | 2.44 | 0.04 | 5.74 | 5.91 |
| Pride × World ID (Empirical) | Low_Mid | 10,269.00 | 6.32 | 2.26 | 0.02 | 6.28 | 6.36 |
| Pride × World ID (Empirical) | Low_High | 1,888.00 | 6.45 | 2.42 | 0.06 | 6.34 | 6.56 |
| Pride × World ID (Empirical) | Mid_Low | 11,695.00 | 6.67 | 2.27 | 0.02 | 6.63 | 6.71 |
| Pride × World ID (Empirical) | Mid_Mid | 54,806.00 | 6.95 | 2.20 | 0.01 | 6.93 | 6.97 |
| Pride × World ID (Empirical) | Mid_High | 10,700.00 | 7.02 | 2.30 | 0.02 | 6.97 | 7.06 |
| Pride × World ID (Empirical) | High_Low | 1,183.00 | 6.88 | 2.26 | 0.07 | 6.75 | 7.01 |
| Pride × World ID (Empirical) | High_Mid | 4,512.00 | 7.15 | 2.05 | 0.03 | 7.09 | 7.21 |
| Pride × World ID (Empirical) | High_High | 2,469.00 | 7.46 | 2.23 | 0.04 | 7.37 | 7.54 |

**Supplemental Table 9.** Pairwise comparisons (Bonferroni/8-adjusted) for each empirical combination

| **Pair** | **Contrast** | **n high x high** | **n other** | **Mean high x high** | **Mean other** | **Difference** | **t** | **df** | **Lower 95% CI** | **Upper 95% CI** | **adjusted-*p*** |
| --- | --- | --- | --- | --- | --- | --- | --- | --- | --- | --- | --- |
| National ID × World ID (Empirical) | High_High - High_Low | 3,339.000 | 1,389.000 | 7.373 | 6.798 | 0.576 | 7.892 | 2,531.316 | 0.433 | 0.719 | <.001 |
| National ID × World ID (Empirical) | High_High - High_Mid | 3,339.000 | 3,640.000 | 7.373 | 7.213 | 0.160 | 3.139 | 6,718.826 | 0.060 | 0.260 | 0.014 |
| National ID × World ID (Empirical) | High_High - Low_High | 3,339.000 | 927.000 | 7.373 | 6.453 | 0.920 | 10.440 | 1,400.619 | 0.747 | 1.093 | <.001 |
| National ID × World ID (Empirical) | High_High - Low_Low | 3,339.000 | 2,757.000 | 7.373 | 6.051 | 1.322 | 21.983 | 5,690.327 | 1.204 | 1.440 | <.001 |
| National ID × World ID (Empirical) | High_High - Low_Mid | 3,339.000 | 8,070.000 | 7.373 | 6.333 | 1.041 | 22.520 | 6,282.108 | 0.950 | 1.131 | <.001 |
| National ID × World ID (Empirical) | High_High - Mid_High | 3,339.000 | 10,791.00 | 7.373 | 6.957 | 0.417 | 9.311 | 5,733.509 | 0.329 | 0.504 | <.001 |
| National ID × World ID (Empirical) | High_High - Mid_Low | 3,339.000 | 11,860.00 | 7.373 | 6.600 | 0.774 | 17.533 | 5,470.044 | 0.687 | 0.860 | <.001 |
| National ID × World ID (Empirical) | High_High - Mid_Mid | 3,339.000 | 57,877.00 | 7.373 | 6.923 | 0.451 | 11.316 | 3,721.738 | 0.373 | 0.529 | <.001 |
| Pride × National ID (Empirical) | High_High - High_Low | 2,628.000 | 352.000 | 7.582 | 6.761 | 0.821 | 6.210 | 430.449 | 0.561 | 1.081 | <.001 |
| Pride × National ID (Empirical) | High_High - High_Mid | 2,628.000 | 5,184.000 | 7.582 | 7.038 | 0.544 | 10.726 | 5,277.141 | 0.445 | 0.643 | <.001 |
| Pride × National ID (Empirical) | High_High - Low_High | 2,628.000 | 664.000 | 7.582 | 6.840 | 0.742 | 7.675 | 980.260 | 0.552 | 0.932 | <.001 |
| Pride × National ID (Empirical) | High_High - Low_Low | 2,628.000 | 4,234.00 | 7.582 | 5.938 | 1.644 | 29.878 | 6,027.224 | 1.536 | 1.752 | <.001 |
| Pride × National ID (Empirical) | High_High - Low_Mid | 2,628.000 | 10,387.00 | 7.582 | 6.319 | 1.263 | 26.811 | 4,342.358 | 1.171 | 1.356 | <.001 |
| Pride × National ID (Empirical) | High_High - Mid_High | 2,628.000 | 5,076.00 | 7.582 | 7.063 | 0.520 | 10.163 | 5,373.239 | 0.419 | 0.620 | <.001 |
| Pride × National ID (Empirical) | High_High - Mid_Low | 2,628.000 | 7,168.00 | 7.582 | 6.452 | 1.130 | 22.985 | 4,950.770 | 1.034 | 1.226 | <.001 |
| Pride × National ID (Empirical) | High_High - Mid_Mid | 2,628.000 | 64,957.00 | 7.582 | 6.957 | 0.625 | 14.810 | 2,866.409 | 0.543 | 0.708 | <.001 |
| Pride × World ID (Empirical) | High_High - High_Low | 2,469.000 | 1,183.00 | 7.456 | 6.882 | 0.575 | 7.223 | 2,299.152 | 0.419 | 0.731 | <.001 |
| Pride × World ID (Empirical) | High_High - High_Mid | 2,469.000 | 4,512.00 | 7.456 | 7.146 | 0.311 | 5.730 | 4,730.832 | 0.205 | 0.417 | <.001 |
| Pride × World ID (Empirical) | High_High - Low_High | 2,469.000 | 1,888.00 | 7.456 | 6.452 | 1.005 | 14.066 | 3,883.803 | 0.865 | 1.145 | <.001 |
| Pride × World ID (Empirical) | High_High - Low_Low | 2,469.000 | 3,128.00 | 7.456 | 5.828 | 1.629 | 26.018 | 5,480.120 | 1.506 | 1.751 | <.001 |
| Pride × World ID (Empirical) | High_High - Low_Mid | 2,469.000 | 10,269.00 | 7.456 | 6.321 | 1.136 | 22.667 | 3,790.513 | 1.037 | 1.234 | <.001 |
| Pride × World ID (Empirical) | High_High - Mid_High | 2,469.000 | 10,700.00 | 7.456 | 7.017 | 0.439 | 8.778 | 3,782.419 | 0.341 | 0.538 | <.001 |
| Pride × World ID (Empirical) | High_High - Mid_Low | 2,469.000 | 11,695.00 | 7.456 | 6.672 | 0.784 | 15.847 | 3,628.136 | 0.687 | 0.881 | <.001 |
| Pride × World ID (Empirical) | High_High - Mid_Mid | 2,469.000 | 54,806.00 | 7.456 | 6.950 | 0.507 | 11.064 | 2,689.291 | 0.417 | 0.597 | <.001 |

Note: adjusted-p refers to Bonferroni-corrected for 8 tests (α/8).
